# Peptide‐Drug Conjugate for Therapeutic Reprogramming of Tumor‐Associated Macrophages in Breast Cancer

**DOI:** 10.1002/advs.202410288

**Published:** 2025-01-22

**Authors:** Anni Lepland, Elisa Peranzoni, Uku Haljasorg, Eliana K. Asciutto, Maria Crespí‐Amer, Lorenzo Modesti, Kalle Kilk, Manuel Lombardia, Gerardo Acosta, Miriam Royo, Pärt Peterson, Ilaria Marigo, Tambet Teesalu, Pablo Scodeller

**Affiliations:** ^1^ Institute of Biomedicine and Translational Medicine University of Tartu Ravila 14B Tartu 50411 Estonia; ^2^ Immunology and Molecular Oncology Diagnostics Veneto Institute of Oncology IOV – IRCCS Padua 35128 Italy; ^3^ Molecular Pathology Research Group Institute of Biomedicine and Translational Medicine University of Tartu Tartu 50411 Estonia; ^4^ Instituto de Ciencias Físicas Universidad Nacional de San Martin (UNSAM) and CONICET Campus Migueletes 25 de Mayo y Francia Buenos Aires CP 1650 Argentina; ^5^ Institute for Advanced Chemistry of Catalonia IQAC‐CSIC Jordi Girona 18–26 Barcelona 08034 Spain; ^6^ Department of biochemistry Institute of Biomedicine and Translational Medicine University of Tartu Ravila 19 Tartu 50411 Estonia; ^7^ Proteomics core facility Centro Nacional de Biotecnologia CNB‐CSIC Calle Darwin 3 Madrid 28049 Spain; ^8^ CIBER‐BBN Networking Centre on Bioengineering Biomaterials and Nanomedicine IQAC‐CSIC Barcelona 08034 Spain; ^9^ Department of Surgery Oncology and Gastroenterology (DISCOG) University of Padova Padova 35128 Italy

**Keywords:** CD206, peptide‐drug conjugate, targeting peptides, triple negative breast cancer, tumor‐associated macrophages

## Abstract

In triple‐negative breast cancer (TNBC), pro‐tumoral macrophages promote metastasis and suppress the immune response. To target these cells, a previously identified CD206 (mannose receptor)‐binding peptide, mUNO was engineered to enhance its affinity and proteolytic stability. The new rationally designed peptide, MACTIDE, includes a trypsin inhibitor loop, from the Sunflower Trypsin Inhibitor‐I. Binding studies to recombinant CD206 revealed a 15‐fold lower K_D_ for MACTIDE compared to parental mUNO. Mass spectrometry further demonstrated a 5‐fold increase in MACTIDE's half‐life in tumor lysates compared to mUNO. Homing studies in TNBC‐bearing mice shows that fluorescein (FAM)‐MACTIDE precisely targeted CD206^+^ tumor‐associated macrophages (TAM) upon intravenous, intraperitoneal, and even oral administration, with minimal liver accumulation. MACTIDE was conjugated to Verteporfin, an FDA‐approved photosensitizer and YAP/TAZ pathway inhibitor to create the conjugate MACTIDE‐V. In the orthotopic 4T1 TNBC mouse model, non‐irradiated MACTIDE‐V‐treated mice exhibited anti‐tumoral effects comparable to those treated with irradiated MACTIDE‐V, with fewer signs of toxicity, prompting further investigation into the laser‐independent activity of the conjugate. In vitro studies using bone marrow‐derived mouse macrophages showed that MACTIDE‐V excluded YAP from the nucleus, increased phagocytic activity, and upregulated several genes associated with cytotoxic anti‐tumoral macrophages. In mouse models of TNBC, MACTIDE‐V slowed primary tumor growth, suppressed lung metastases, and increased markers of phagocytosis and antigen presentation in TAM and monocytes, increasing the tumor infiltration of several lymphocyte subsets. MACTIDE‐V is proposed as a promising peptide‐drug conjugate for modulating macrophage function in breast cancer immunotherapy.

## Graphical abstract

The peptide‐drug conjugate “MACTIDE‐V” targets and reprograms tumor‐associated macrophages (TAM) suppressing metastases in highly metastatic triple‐negative breast tumors in mice. MACTIDE‐V is based on a new stable and orally available CD206‐binding peptide (MACTIDE), and the small molecule drug Verteporfin (V). MACTIDE‐V acts on the macrophage YAP/TAZ pathway promoting a phenotype associated with antigen presentation, phagocytosis, and immunostimulation.

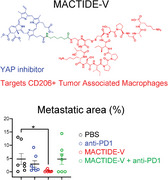

## 1 Introduction

Established solid tumors favor immunosuppressive and angiogenic phenotypes of macrophages that induce progression and metastasis. By secreting cytokines that attract and skew macrophage functions, most tumors can expand the pro‐tumoral tumor‐associated macrophages (TAM) population, making it the most prominent immune cell type of the tumor microenvironment. Pro‐tumoral TAM are important in triple negative breast cancer (TNBC)^[^ ^1^ ^]^ where antibody‐dependent cellular cytotoxicity‐based therapies are currently not available due to a lack of specific cancer cell receptors. Tackling the tumor‐promoting microenvironment through targeting TAM is therefore an emerging alternative.

To target TAM, we previously developed the mUNO targeting peptide^[^ ^2^, ^3^ ^]^ (sequence: CSPGAK), which binds to the mannose receptor CD206, over‐expressed in a subset of pro‐tumoral TAM.^[^ ^4^ ^]^ In the TNBC 4T1 model, we demonstrated that mUNO specifically targeted TAM with minimal accumulation in the liver.^[^ ^5^ ^]^

Targeting CD206 is also promising for diagnostic purposes, as an increased number of CD206^+^ cells in lymph nodes correlates with relapse onset in some cancers.^[^ ^6^ ^]^ Most synthetic compounds designed to target CD206 utilize mannose as the recognition moiety. However, since mannose exhibits low affinity in the millimolar range,^[^ ^7^ ^]^ achieving effective binding requires multivalent presentation.^[^ ^8^ ^]^

Short linear targeting peptides are selective ligands capable of directing therapeutic or imaging cargos to tumors.^[^ ^9^ ^]^

However, their unconstrained structure results in high conformational flexibility, leading to low binding affinity and susceptibility to proteolytic degradation. While their relatively low affinity can be advantageous for multivalent ligand applications,^[^ ^10^ ^]^ developing monovalent peptide‐drug conjugates (PDCs) requires targeting moieties with enhanced affinity and stability.

Here, we engineered mUNO to enhance its affinity and proteolytic stability for use in a monovalent format.

To achieve this, we leveraged the structural advantages of the Sunflower Trypsin Inhibitor I (SFTI‐1), a conformationally constrained, plant‐derived peptide, featuring two loops bridged by a disulfide bond. The primary loop of SFTI‐1 inhibits serine proteases, including trypsin and pepsin, while the cyclization loop has been successfully modified by other groups to incorporate foreign peptides without compromising enzyme inhibition or oral bioavailability.^[^ ^11^ ^]^ Moreover, SFTI‐1 variants with an open cyclization loop have demonstrated trypsin inhibition constants comparable to the intact structure.^[^ ^12^, ^13^ ^]^

We report here a new CD206‐binding peptide, MACTIDE, which demonstrates superior affinity and stability compared to its predecessor and is suitable for oral delivery. Additionally, we present a PDC, MACTIDE‐Verteporfin (MACTIDE‐V), with dual therapeutic applications. First, it enables light‐dependent depletion of CD206^+^ macrophages via PDT. Second, it reprograms TAM toward an anti‐tumoral phenotype through the action of Verteporfin, a YAP/TAZ pathway inhibitor.

## 2 Results

### 2.1 Design and Molecular Dynamics of MACTIDE

**Figure 1:**
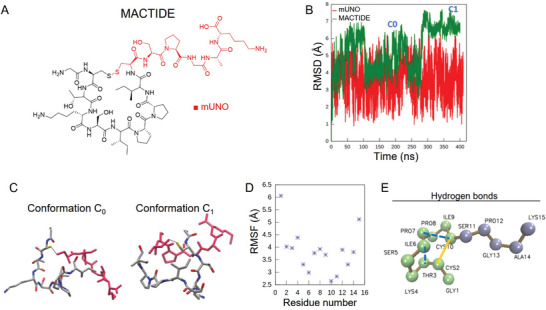
Design and molecular dynamics of MACTIDE. A) MACTIDE structure with the CD206‐binding motif mUNO in red. B) RMSD to the average structure for heavy atoms of mUNO (red lines) and MACTIDE (green line), in two 400 ns molecular dynamics for mUNO and MACTIDE in solution. C) The two most populated conformations of MACTIDE, C0, and C1, with the mUNO motif in red. D) RMSF of each residue for MACTIDE in solution. E) Intra hydrogen bonds in MACTIDE (dashed blue lines), between Pro7‐Cys10 with an occupancy of 75% and between Ile6‐Thr3 with an occupancy of 10% (present in the C1 conformation); the yellow line indicates the disulfide bond.

To design MACTIDE, we inserted mUNO (sequence CSPGAK) instead of the cyclization loop of SFTI‐1. Then, we removed the head‐to‐tail cyclization to accommodate a fluorophore/drug on the N‐terminus. To increase flexibility between the cyclization loop and the fluorophore/drug, we added glycine G1 (**Figure** **1**A), the amino acid with the highest flexibility.^[^ ^14^ ^]^ To assess if our modifications constrained the mUNO motif in MACTIDE, we performed computational analyses to study its structure in solution.

An ensemble of MACTIDE conformations in solution was generated through an all‐atom molecular dynamic simulation. The ensemble was clustered using a root mean square deviation (RMSD) criterion to group conformations with similar structural arrangements and identify the representative conformation. MACTIDE predominantly adopted two configurations, C0 and C1, with variations of ≈1 Å from their centroid structure (Figure 1B, green). In contrast, mUNO exhibited greater variation during simulations, with deviations reaching 3 Å from its average structure (Figure 1B, red), indicating that MACTIDE is significantly more constrained than mUNO. The C0 and C1 configurations of MACTIDE were present in 44% of the trajectory and their structures are shown in Figure 1C.

We also assessed the rigidity of MACTIDE by analyzing the root mean square fluctuation (RMSF) of each residue (Figure 1D). Only the two terminal residues exhibited greater flexibility, while the rest of the peptide showed similar RMSF values. We later calculated the intra‐hydrogen bonds in MACTIDE (Figure 1E, dashed blue lines). Both conformations exhibited a moderate hydrogen bond (mainly electrostatic) between Cys10 and Pro7, with an occupancy of 75%. The C1 conformation presented an additional hydrogen bond between Ile6 and Thr3. The primary loop of acyclic SFTI‐1 only presents the hydrogen bond Thr3‐Ile9,^[^ ^12^ ^]^ which was not observed in our structure, indicating that the structure of the primary loop in MACTIDE differs from that in acyclic SFTI‐1.

### 2.2 Docking Reveals High Binding Energy of MACTIDE to CD206 and Ligand‐Induced Conformational Change

**Figure 2:**
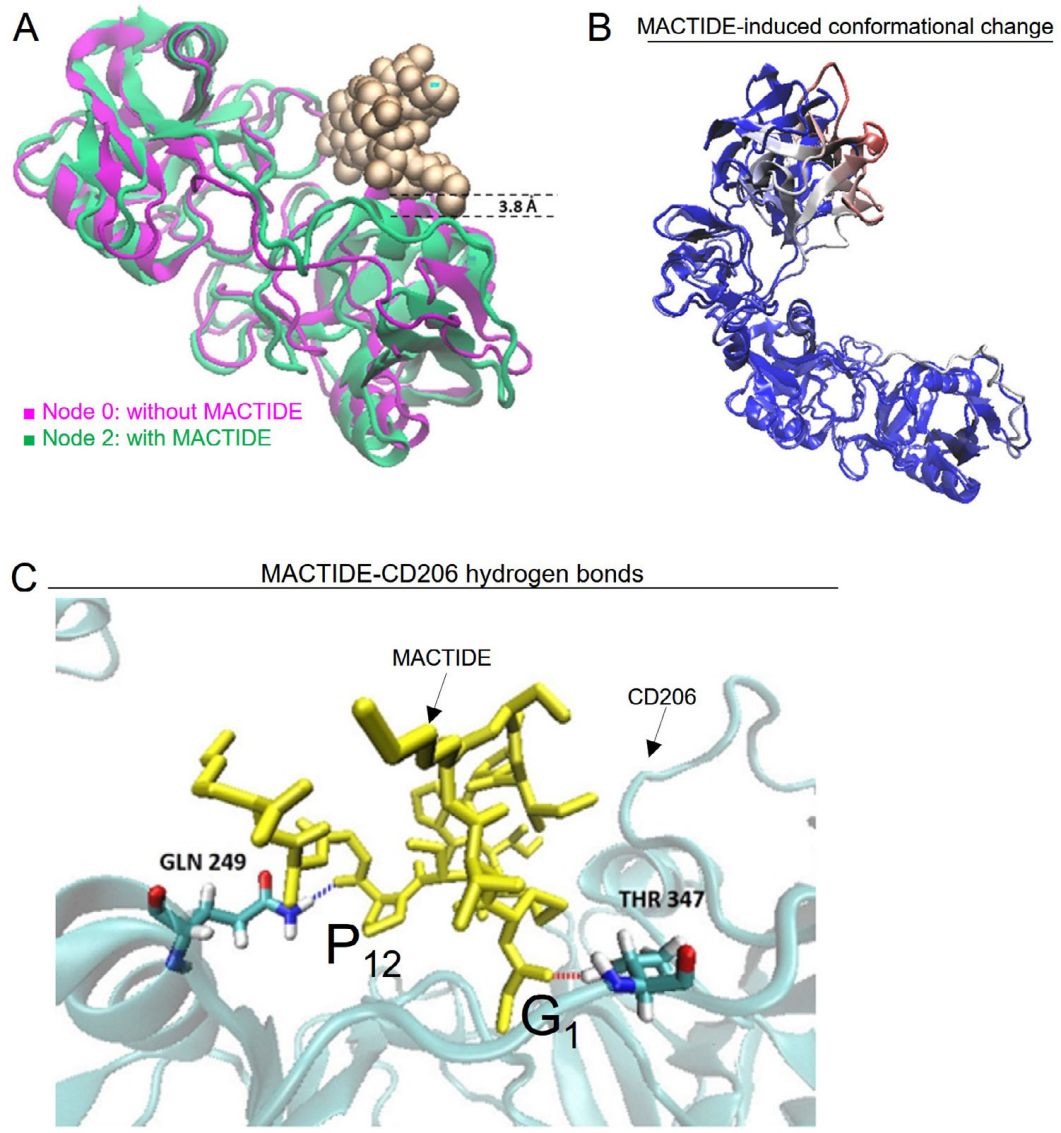
Docking shows high binding energy of MACTIDE to CD206 and ligand‐induced conformational change. A) CTLD2 domain for cluster node 2 (green) compared with cluster node 0 (purple). MACTIDE is represented as VDW golden spheres. A large alpha helix displacement of 3.8 Å was found in node 2 making space for the peptide to bind. B) Node 2 compared to the crystal structure 5XTS and colored by RMSD. Significant differences (in red) were found in the CysR domain. C) Hydrogen bonds (dashed blue and red lines) formed at the docking pose, where MACTIDE is shown in yellow and CD206 in cyan.

To estimate the binding site of MACTIDE, we utilized HPEPDOCK,^[^ ^15^ ^]^ a hierarchical algorithm for blind and flexible peptide docking. The flexibility of CD206 was addressed through prior simulation of the receptor in solution, followed by clustering to identify the most populated receptor conformations (for more details see Materials and Methods). The best docking score was found for a receptor configuration where the alpha helix at CTLD2, defined by Thr360‐Tyr373, is displaced downward by 3.8 Å, creating space for the peptide to accommodate (**Figure** **2**A). In the peptide‐bound configuration, we noted slight differences in the CysR region and closing of the V‐shaped portion of the receptor compared to the crystal structure PDB (5XTS) (Figure 2B). We previously documented a similar ligand‐induced conformational change in CD206 when bound to mUNO.^[^ ^3^ ^]^ The docking pose was located in the region between lectin domains CTLD1‐2, the same region that binds mUNO,^[^ ^3^ ^]^ with MACTIDE establishing closest contacts with Asp273 and Thr324. These simulations revealed hydrogen bonds between G1‐Thr 347 and P12 ‐Gln 249 (Figure 2C) and the mUNO motif within MACTIDE (C10‐K15) pointed toward the receptor. The HPEPDOCK docking score of MACTIDE was considerably higher than the one of mUNO against the same receptor: −181.7 versus −115.0, respectively. Moreover, since G1 participated in a hydrogen bond, to investigate its contribution we performed docking without it; this revealed a lower docking score of −172.5, suggesting that G1 not only serves as a flexible spacer but also contributes to binding. Based on this data, we decided to synthesize MACTIDE and evaluate it experimentally.

### 2.3 MACTIDE Shows Enhanced Affinity and Proteolytic Stability

**Figure 3:**
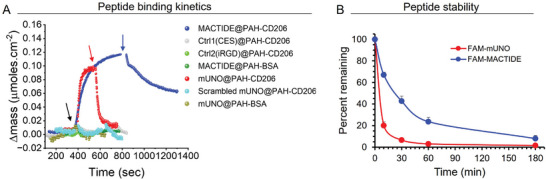
MACTIDE has higher affinity and proteolytic stability than mUNO. A) QCM experiment of MACTIDE, mUNO, and control peptides at a final concentration of 10 µm in PBS on multilayers of PAH/CD206 or control multilayers of PAH/BSA. The black arrow indicates when the peptides were added, blue and red arrows indicate when the washing step with PBS started. B) Integrity of FAM‐MACTIDE and FAM‐mUNO measured by LC‐MS at different time points after incubation of peptides with lysate derived from a 4T1 tumor.

To confirm the capacity of MACTIDE to bind to CD206, we evaluated binding to recombinant CD206 included in a viscoelastic film, using Quartz Crystal Microbalance (QCM). QCM is a powerful technique used to study label‐free ligand‐receptor interactions in solution,^[^ ^16^, ^17^, ^18^, ^19^ ^]^ wherein peptide binding and dissociation are sensed through a mass increase or decrease on a resonating crystal surface functionalized with the receptor. Here, we evaluated the mass changes of MACTIDE when binding and dissociating in solution to CD206 immobilized on a viscoelastic film,^[^ ^20^ ^]^ deposited on a gold‐modified quartz crystal resonating at 5 MHz. Recombinant CD206 was immobilized using layer‐by‐layer (LBL) self‐assembly, electrostatically‐driven adsorption of charged polyelectrolytes on a layer of polyallylamine (PAH).^[^ ^21^, ^22^, ^23^ ^]^ These experiments showed that MACTIDE bound to PAH/CD206 (**Figure** **3**A, blue) and incompletely and slowly dissociated upon washing with PBS (Figure 3A, blue arrow), whereas two control peptides did not bind to the same multilayer (Figure 3A, gray and light green). Additionally, MACTIDE did not bind to the control multilayer PAH/BSA (Figure 3A, dark green). The mUNO peptide also showed binding to the PAH/CD206 multilayer but with complete and faster dissociation kinetics than MACTIDE upon washing with PBS (Figure 3A, red), whereas the scrambled mUNO peptide did not bind to the same multilayer (Figure 3A, cyan) and mUNO did not bind to the control multilayer PAH/BSA (Figure 3A, brown). Fitting the association and dissociation curves revealed that the constant of association was slightly faster for mUNO (6.5 × 10^2^ versus 14 × 10^2^ M^−1^ s^−1^), but the main difference was in the dissociation constant, which was 30‐fold slower for MACTIDE (2.5 × 10^−4^ versus 8.7 × 10^−3^ s^−1^), resulting in K_D_ = 0.38 µm for MACTIDE and K_D_ = 6 µm for mUNO.

We next evaluated the stability of fluorescein (FAM)‐MACTIDE against proteases from breast tumors. To this end, we incubated both FAM‐MACTIDE and FAM‐mUNO with a tumor lysate obtained from 4T1 orthotopic TNBC tumors using different timepoints and evaluated the integrity of the peptide using liquid chromatography‐mass spectrometry (LC‐MS). We observed that the half‐life of FAM‐MACTIDE in this tumor lysate increased 5‐fold with respect to FAM‐mUNO (Figure 3B).

### 2.4 FAM‐MACTIDE Targets CD206^+^ TAM Using Different Administration Routes

To show that MACTIDE can be used to deliver a conjugated payload to CD206^+^ TAM, we administered FAM‐MACTIDE to mice bearing 4T1 tumors, using different administration routes. We previously reported high CD206^+^ TAM infiltration in this tumor model.^[^ ^2^ ^]^

The targeting efficacy was evaluated by immunostaining of tumor sections for FAM and CD206.

**Figure 4:**
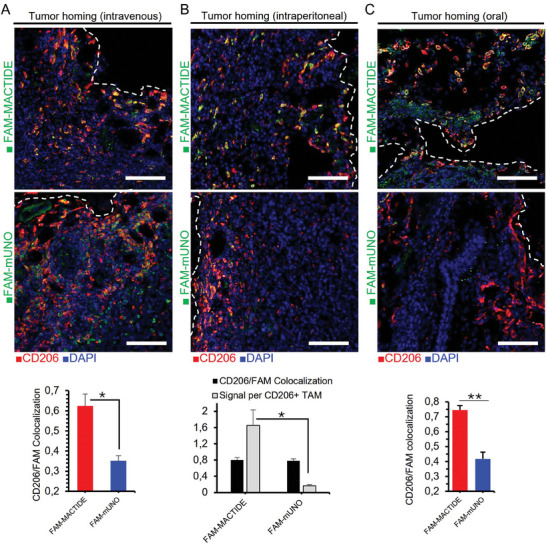
FAM‐MACTIDE targets CD206^+^ TAM using different administration routes. Thirty nmol of FAM‐MACTIDE or FAM‐mUNO were administered i.v. (A), i.p. (B), or orally (C) and left to circulate for 24 h. After 24 h, mice were sacrificed, and the organs were collected, fixed, cryoprotected, sectioned, and immunostained for FAM (shown in green) and CD206 (shown in red). The CD206/FAM colocalization indices were calculated from representative images from *n* = 3 tumors, using Fiji (Mandler's tM2 index) and the signal intensity per CD206^+^ TAM (i.p. administration) was quantified using ImageJ (B). Scale bars = 100 µm. ^*^ *p* ≤ 0.05, ^**^ *p* ≤ 0.01 (Anova one‐way fisher LSD).

FAM‐MACTIDE showed CD206^+^ TAM targeting when administered intravenously (i.v.), whereas low CD206^+^ TAM targeting was observed for FAM‐mUNO using the same route (**Figure** **4**A). With intraperitoneal (i.p.) administration, both FAM‐MACTIDE and FAM‐mUNO showed high CD206/FAM colocalization (80%) (Figure 4B), but FAM‐MACTIDE showed a 10‐fold higher FAM intensity per CD206^+^ TAM. FAM‐MACTIDE also targeted CD206^+^ TAM when administered orally (Figure 4C). We subsequently determined the blood half‐life of FAM‐MACTIDE using i.v. and i.p. administration (Figure S1, Supporting Information). As these values are equal to those obtained previously for FAM‐mUNO,^[^ ^5^ ^]^ we propose that the higher affinity and proteolytic stability of MACTIDE account for the homing differences observed between the two peptides. Importantly, we observed low liver accumulation with these administration routes for both peptides (Figure S2, Supporting Information).

### 2.5 A MACTIDE‐Verteporfin (MACTIDE‐V) Conjugate has Light‐Dependent and Light‐Independent In Vivo Activity in a Breast Cancer Model

**Figure 5:**
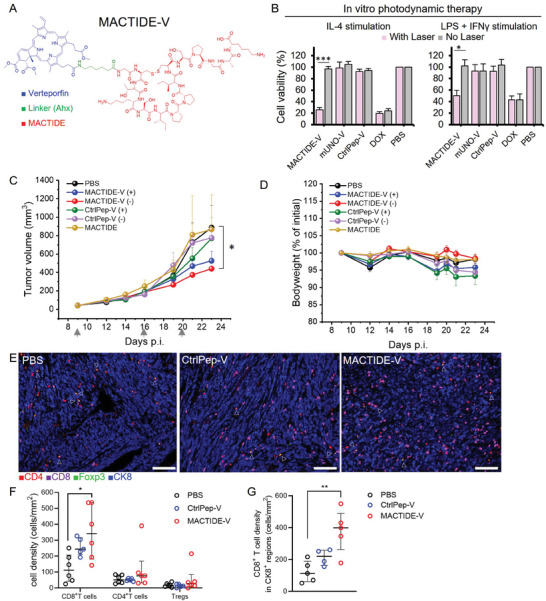
In vitro and in vivo photodynamic therapy with MACTIDE‐V. A) Structure of MACTIDE‐V. B) MACTIDE‐V, CtrlPep‐V, and DOX were incubated with primary human macrophages (obtained from monocytes derived from human blood buffy coat) at 30 µm for 1 h at 37 °C, followed by 2 washes with media. Then, the groups shown in pink were irradiated with 10 J cm^−2^ (irradiance: 170 mW cm^−2^, spot diameter: 0.5 cm). Then, the cells were incubated for 48 h and the cell viability was determined using the MTT((3‐(4,5‐Dimethylthiazol‐2‐yl)‐2,5‐Diphenyltetrazolium Bromide) assay. Here, the graph shows the average of *n* = 6 experiments. C) Treatment with MACTIDE‐V and CtrlPep‐V with and without laser (denoted by + and −, respectively), in mice bearing orthotopic 4T1 tumors (*n* = 6/group) in Balb/C. Peptide‐V conjugates were administered i.p. at a dose of 30 nmol (1 mg Kg^−1^ in Verteporfin). For the irradiated groups, 4 h after administration of conjugates or PBS, the mice were irradiated with 100 J cm^−2^; shown is primary tumor volume progression during treatment. Gray arrows indicate injection days. D) Bodyweight during the treatment as % of the initial one. E) Representative mIHC images of PBS, CtrlPep‐V, and MACTIDE‐V‐treated tumors, 20X magnification, scale bar = 100 µm, white triangles point to Foxp3^+^ cells. F) CD8^+^ T cells, CD4^+^ T cells, and Treg density, *n* = 6 mice/group, with every dot representing the mean cell density of each tumor obtained from 20 images. G) CD8^+^ T cells density in CK8^+^‐dense tumor regions. *n* = 5 mice/group, with every dot representing the mean cell density of each tumor obtained from 20 images. Median ± interquartile range. One‐way ANOVA with multiple comparisons, ^*^ *p* ≤ 0.05, ^**^ *p* ≤ 0.01.

We next coupled MACTIDE to the photosensitizer Verteporfin, intending to deplete CD206^+^ TAM using photodynamic therapy (PDT). To this end, carboxy‐Verteporfin was coupled to the N‐terminus of MACTIDE, a construct referred to as “MACTIDE‐V” (**Figure** **5**A). We performed in vitro photodynamic therapy with MACTIDE‐V on primary human macrophages stimulated with IL‐4 or LPS + IFNγ. Irradiated MACTIDE‐V depleted 80% of IL‐4‐stimulated macrophages and 40% of LPS + IFNγ‐stimulated macrophages (we previously showed that LPS + IFNγ‐stimulated macrophages express CD206 at lower levels^[^ ^24^ ^]^). The non‐irradiated MACTIDE‐V showed no toxicity to these two cell types. Doxorubicin (DOX), a chemotherapeutic drug routinely used in clinic,^[^ ^25^ ^]^ showed similar and irradiation‐independent toxicity. The control conjugates mUNO‐V and CtrlPep‐V showed no toxicity in any of the cells regardless of irradiation (Figure 5B).

Prompted by the consistent laser‐induced and preferential toxicity of MACTIDE‐V on IL‐4‐stimulated macrophages, we decided to evaluate its in vivo therapeutic effect on mice survival in the orthotopic 4T1 model. Although no significant differences were observed in mice survival (Figure S3, Supporting Information), MACTIDE‐V followed by tumor irradiation slowed down the tumor growth compared to the control groups PBS (+), CtrlPep‐V (‐), CtrlPep‐V (+) and MACTIDE (‐) (“+” denotes irradiated and “‐” denotes non‐irradiated), although these differences did not reach statistical significance. Interestingly, it was MACTIDE‐V (‐) that showed the strongest tumor volume reduction, significant respect to PBS (Figure 5C). Moreover, MACTIDE‐V (‐) exerted a negligible effect on body weight (Figure 5D). This prompted us to further investigate the effect of MACTIDE‐V (‐), which hereafter will be denoted simply as “MACTIDE‐V”. To obtain further information on the effect of MACTIDE‐V in this tumor model, we investigated T cell infiltration in treated tumors, using multiplex immunohistochemistry (mIHC), comparing MACTIDE‐V (‐) group to PBS and CtrlPep‐V (‐) groups. The mIHC analysis showed a significantly higher infiltration of CD8^+^ T cells in the MACTIDE‐V group and no differences in the density of regulatory T cells (Tregs) (Figures 5E,F). Importantly, the higher CD8^+^ T cell density was also observed in cytokeratin 8 (CK8^+^)‐dense regions (Figure 5G), highlighting the proximity of effector cells to cancer cells, crucial for anti‐tumor cytotoxicity.

### 2.6 MACTIDE‐V Excludes YAP from the Nucleus and Increases Phagocytosis and Anti‐Tumoral Gene Expression in Mouse Bone Marrow‐Derived Macrophages

**Figure 6:**
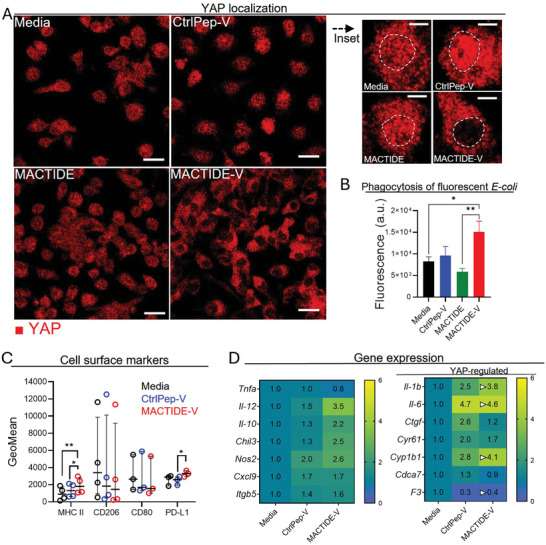
MACTIDE‐V promotes a YAP‐ mediated anti‐ tumoral switch in bone marrow‐derived macrophages. Day 5 BMDM were incubated for 4 h with 10 µm conjugates, washed, and followed‐up in media. A) YAP Immunofluorescence (shown in red) was analyzed 3 h after treatment and B) phagocytosis of fluorescent *E. coli* particles at 48 h follow‐up. C) Flow cytometry on BMDM 48 h after treatment. Left panel, GeoMean of MHC II, CD206, CD80, and PD‐L1 in Balb/c BMDM. Median ± interquartile range. Repeated measures one‐way ANOVA with multiple comparisons, for *n* = 4 independent experiments. ^*^ *p* ≤ 0.05, ^**^ *p* ≤ 0.01. D) Heatmap of the mRNA expression of genes involved in the functional activation of BMDM, YAP signaling, and adhesion, measured by real‐time PCR 48 h after treatment in BALB/c BMDM. *n* = 3 independent experiments.

We speculated that the observed in vivo effect of MACTIDE‐V could be mediated by the modulation of macrophage phenotype elicited by Verteporfin, a drug that inhibits the co‐transcription factor Yes Associated Protein (YAP), and prevents the anti‐inflammatory shift in macrophages.^[^ ^26^ ^]^ We therefore analyzed the effect of MACTIDE‐V on YAP localization and phenotypic changes in mouse bone marrow‐derived macrophages (BMDM) in vitro. We first treated BMDM with MACTIDE‐V, MACTIDE, or CtrlPep‐V and evaluated changes in YAP localization 3 h later using confocal microscopy. MACTIDE‐V excluded YAP from the nucleus, whereas in the other groups, YAP appeared uniformly spread throughout the cell (**Figure** **6**A). These observations are consistent with the reported effect of Verteporfin sequestering YAP in the cytoplasm.^[^ ^27^, ^28^ ^]^

We then analyzed if MACTIDE‐V affected the phagocytic activity of BMDM in vitro. Phagocytosis analysis of fluorescently labeled *E. coli* bacteria showed that MACTIDE‐V treated BMDM presented significantly higher phagocytosis than MACTIDE treated and untreated BMDM (Figure 6B). Moreover, investigation of phagocytosis of live 4T1 cancer cells labeled with green fluorescent protein (GFP), revealed cancer cell phagocytosis only in the MACTIDE‐V treated BMDM, with negligible phagocytosis in CtrlPep‐V, MACTIDE and untreated BMDM (Figure S4, Supporting Information).

We previously demonstrated that free Verteporfin is not cell‐specific,^[^ ^29^ ^]^ and others showed it elicits cardiotoxicity in vivo;^[^ ^30^ ^]^ for these reasons in this study, we chose to target Verteporfin using MACTIDE. At the concentrations used in the present study (10 µm), Verteporfin alone was highly toxic to BMDM (Figure S5, Supporting Information). For the reasons above and because we observed no effects with free MACTIDE, subsequent experiments focused on MACTIDE‐V and CtrlPep‐V.

We subsequently analyzed the expression of different markers of BMDM, both at the protein and the mRNA level, 48 h after treatment with CtrlPep‐V and MACTIDE‐V. In Balb/C BMDM, MACTIDE‐V upregulated the expression of class II major histocompatibility complex (MHC II) and PD‐L1 compared to CtrlPep‐V and untreated conditions, with no significant changes in CD206 and CD80 expression (Figure 6C). At the mRNA level, MACTIDE‐V induced a clear increase in *Il‐12*, *Chil3 and Nos2* genes, (Figure 6D, left panel). In BMDM obtained from C57/Bl6 (B6) mice, MACTIDE‐V increased MHCII expression and upregulated the inflammatory genes *Il‐1b, Il‐12* and *Nos2* (Figure S6, Supporting Information). To further investigate whether the effect of MACTIDE‐V was mediated by YAP targeting, we analyzed a set of 7 YAP‐regulated genes (Figure 6D, right panel). These results showed that of the 14 genes analyzed, the largest changes occurred in YAP‐regulated genes Il‐1b, Il‐6, Cyp1b1, and F3 (indicated by white triangles) and were in line with YAP inhibition,^[^ ^31^, ^32^ ^]^ while the remaining three YAP‐regulated genes Ctgf, Cyr61 and Cdca7 did not show appreciable changes. These results, together with the nuclear YAP exclusion observed, indicate that the action of MACTIDE‐V is mediated by YAP inhibition.

### 2.7 MACTIDE‐V Exerts Anti‐Tumoral and Anti‐Metastatic Effect in Orthotopic 4T1.2

**Figure 7:**
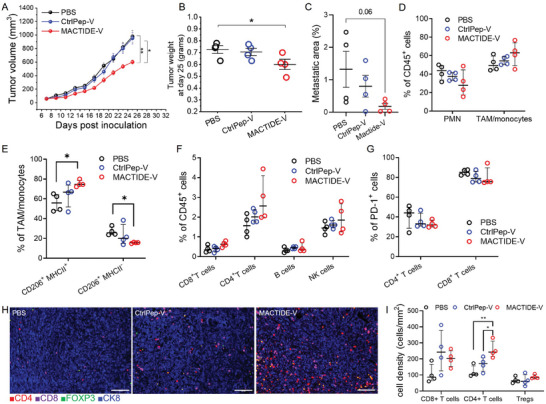
MACTIDE‐V slows tumor growth and suppresses lung metastasis in orthotopic 4T1.2. 4T1.2 bearing mice were treated with 9 doses of CtrlPep‐V, MACTIDE‐V (1 mg Kg^−1^ in Verteporfin per dose), or PBS i.p. every other day, while monitoring the primary tumor volume (A). Mice were sacrificed on day 25, tumor weights were measured (B) and pulmonary metastasis areas were quantified from H&E sections (C). D–G) Tumor cell suspensions were analyzed by flow cytometry (*n* = 4 per group). H) Representative mIHC images of PBS, CtrlPep‐V, and MACTIDE‐V treated tumors, scale bar = 100 µm. I) CD8^+^ T cells, CD4^+^ T cells, and Treg density obtained from mIHC images (*n* = 6 mice/group), with every dot representing the mean cell density of each tumor obtained from 20 images. Median ± interquartile range. One‐way ANOVA with multiple comparisons, ^*^ *p* ≤ 0.05, ^**^ *p* ≤ 0.01.

We evaluated the effect of MACTIDE‐V on tumor progression and metastasis in the highly metastatic orthotopic 4T1.2 model.^[^ ^33^ ^]^ When tumors reached 55 mm^3^ we began the treatment with MACTIDE‐V or CtrlPep‐V every other day until day 23, using the same dose used in section 5. 4 weeks after tumor induction, MACTIDE‐V‐treated mice showed significantly smaller tumor volumes compared to CtrlPep‐V and PBS (**Figure** **7**A), as well as smaller endpoint tumor weights (Figure 7B) and fewer pulmonary metastasis (Figure 7C). No body weight loss was detected in any of the groups (Figure S7, Supporting Information).

We then analyzed the immune cell populations of the tumor microenvironment by flow cytometry. No effects were observed on the polymorphonuclear (PMN) or TAM/monocyte cell populations (Figure 7D). MACTIDE‐V increased the percentage of MHCII^+^CD206^+^ TAM/monocytes and decreased the MHC II^−^CD206^+^ subset (Figure 7E).

In addition, MACTIDE‐V treatment induced a rise in the proportion of tumor‐infiltrating T cells and NK cells with respect to CtrlPep‐V and PBS, but no change in B cells (Figure 7F). We also noticed a decrease in PD‐1 expression in both CD8^+^ and CD4^+^ T cells in MACTIDE‐V‐treated tumors (Figure 7G), possibly underlying a reduction in exhausted T cells.^[^ ^34^ ^]^

The mIHC analysis of 4T1.2 tumors revealed a significant increase in CD4^+^ T cell density in the MACTIDE‐V‐treated group compared to CtrlPep‐V and PBS, an increasing trend in CD8^+^ T cell density with both conjugates and no differences in Treg density (Figure 7H,I). These data demonstrate that MACTIDE‐V can promote an anti‐tumoral TAM/monocyte phenotype, which is accompanied by an influx of effector cells in the tumor.

The receptor of MACTIDE‐V, CD206, can also be expressed on dendritic cells. However, in our models, 90% of CD206^+^ cells in the tumor microenvironment are F4/80‐positive cells (Figure S8, Supporting Information), i.e., belonging to the monocyte‐macrophage lineage, suggesting that the main biological impact of CD206‐targeting derives prevalently from targeting TAM rather than dendritic cells.

To investigate potential off‐target risk in vivo caused by MACTIDE‐V we analyzed the serum levels of the hepatic enzyme alanine aminotransferase (ALAT) following a 3 mg Kg^−1^ dose of MACTIDE‐V in healthy mice. If MACTIDE‐V causes off‐target inflammation, it would likely occur in the liver (the organ that expresses the MACTIDE receptor) and would reflect on ALAT levels (liver inflammation produces a > 2‐fold increase in ALAT^[^ ^35^ ^]^). Our data showed that MACTIDE‐V did not increase the ALAT levels (Table S1, Supporting Information), which agrees with the observed absence of MACTIDE accumulation in the liver. Additionally, MACTIDE‐V showed no signs of overt renal toxicity as evidenced by the Creatinine levels (Table S1, Supporting Information). To investigate possible inflammation‐associated risk in humans, we quantified the secretion of TNF‐α and IFN‐γ – two macrophage‐secreted cytokines strongly implicated in systemic inflammation^[^ ^36^ ^]^ – in cultured primary human macrophages treated with MACTIDE‐V. MACTIDE‐V did not significantly increase TNF‐α or IFN‐γ, it significantly increased the inflammatory cytokines IL‐12 and IL‐23 and significantly decreased the anti‐inflammatory cytokines IL‐1RA (Figure S9, Supporting Information).

### 2.8 MACTIDE‐V Treated Tumors do not Benefit from Concomitant PD‐1 Blockade

**Figure 8:**
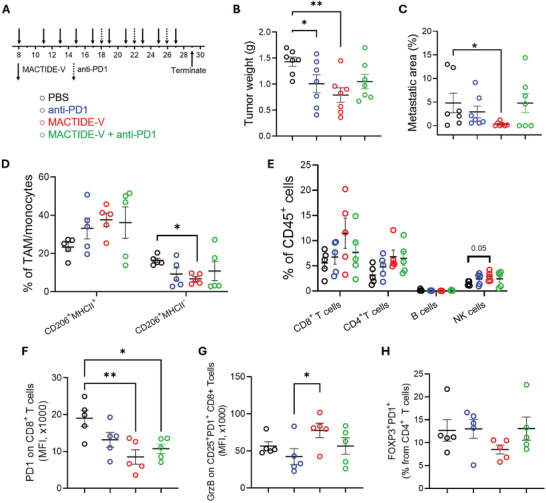
MACTIDE‐V‐treated tumors do not benefit from concomitant anti‐PD‐1 blockade in orthotopic 4T1.2. 4T1.2 bearing mice were treated with 9 doses of CtrlPep‐V, MACTIDE‐V (1 mg Kg^−1^ in Verteporfin per dose), or PBS i.p. every other day according to the scheme in (A). Anti‐PD‐1 injections started 16 days post‐tumor induction (three injections of 200 µg each). Mice were sacrificed on day 29, their tumor weights were analyzed (B), pulmonary metastases area quantified from H&E‐stained sections (C), and their tumors were analyzed with flow cytometry (D–H).

To understand whether the treatment with MACTIDE‐V could render 4T1.2 tumors more sensitive to PD‐1 immune checkpoint blockade (currently used in TNBC patients in combination with chemotherapy), we performed a treatment study with MACTIDE‐V, anti‐PD‐1, or a combination of both, according to the scheme shown in **Figure** **8**A.

Both MACTIDE‐V and anti‐PD‐1 blockade significantly reduced the primary tumor mass, but MACTIDE‐V had a superior effect on reducing pulmonary metastases. Interestingly, no synergy was observed in the combination therapy in this setting (Figures 8B,C). None of the experimental groups exhibited significant bodyweight loss (Figure S10, Supporting Information).

Analysis of the tumor microenvironment by flow cytometry showed that MACTIDE‐V elicited the highest increase in the CD206^+^MHCII^+^ population and the highest decrease in the CD206^+^MHCII^−^ population of TAM/monocytes (Figure 8D). MACTIDE‐V also significantly increased the CD86^+^MHCII^+^ population and decreased the CD86^−^MHCII^−^ population of TAM/monocytes (Figure S11, Supporting Information).

MACTIDE‐V generated the highest increase in CD4^+^ T cells, CD8^+^ T cells and NK cells, although these differences reached statistical significance only for NK cells, with no differences in B cell infiltration (Figure 8E). As already observed in the previous experiments, MACTIDE‐V reduced the expression of PD‐1 on CD8^+^ T cells (Figure 8F) and also increased the expression of Granzyme B (GrzB) in CD25^+^PD‐1^+^ CD8^+^ T cells (Figure 8G). A decreasing trend in the immunosuppressive FOXP3^+^PD‐1^+^ population among CD4^+^ T cells was observed in the MACTIDE‐V treated group compared to the other experimental conditions (Figure 8H).

These data suggest that MACTIDE‐V can modulate TAM/monocytes by inducing antigen presentation and co‐stimulation functions, accompanied by an increase in effector cells with stronger cytotoxic potential.

## 3 Discussion

We designed a CD206‐targeting peptide, MACTIDE, displaying higher affinity, stability, and oral activity than its predecessor peptide mUNO. The improved affinity of MACTIDE allowed us to develop a potent monovalent PDC, something which we did not achieve using mUNO as a targeting peptide. Monovalent PDCs are attractive and translationally relevant drug candidates because they bypass the need for synthetically complex and multiparametric designs, such as nanoparticles or multivalent systems. Peptides and PDCs are becoming increasingly popular for cancer therapy, owing to their selectivity and high penetration in solid tumors.^[^ ^37^, ^38^ ^]^ The other class of commonly used targeting ligands, antibodies, suffer from poor penetration in some solid tumors, and antibody‐drug conjugates have shown toxicity‐related limitations.^[^ ^39^ ^]^

MACTIDE showed no hepatic accumulation, an organ known to express its receptor, CD206. We previously showed that 4T1 tumors are leaky and express higher levels of CD206 than the liver.^[^ ^24^ ^]^ Moreover, blood residence time is known to be higher in tumors.^[^ ^40^ ^]^ Given the slow binding kinetics of MACTIDE to CD206, the higher residence time of MACTIDE in the tumor and the higher expression of its receptor there, likely contribute to the tumor selectivity observed.

Verteporfin (V), is a photosensitizer and inhibitor of the activation of YAP.^[^ ^41^ ^]^ Our PDC, MACTIDE‐V, was able to solubilize Verteporfin, which would otherwise need to be formulated in liposomes or be given with dimethyl sulfoxide as it is insoluble in water. Verteporfin, in its liposomal formulation Visudyne, is an approved drug for certain ophthalmological indications. In clinical trials for cancer therapy, Verteporfin is mostly used for photodynamic therapy (clinical trial identifiers: NCT03067051, NCT03033225, NCT04590664).

Our initial aim was to deplete TAM using photodynamic therapy, but to our surprise, non‐irradiated MACTIDE‐V treated mice experienced a similarly potent anti‐tumoral effect in vivo than those treated with irradiated MACTIDE‐V. Based on this observation, we focused subsequent investigations on non‐irradiated MACTIDE‐V, revealing that this particular conjugate alters the TAM phenotype.

Preclinical and clinical studies^[^ ^24^, ^42^, ^43^ ^]^ have shown that depletion of TAM does not always produce a strong anti‐tumor response. TAM depletion might not be the most adequate approach to modulate the tumor microenvironment as it could eliminate protective, sometimes anti‐tumoral, cell populations. The modulation of TAM phenotype, on the other hand, appears to be a more promising approach, as suggested by other studies^[^ ^44^, ^45^ ^]^ and supported by the results presented here.

Our studies indicate that treatment with MACTIDE‐V promoted a TAM/monocyte phenotype associated with phagocytosis and antigen uptake and presentation, induced CD4^+^ or CD8^+^ T cell infiltration without Treg increase, and induced cytotoxicity markers in CTLs. Moreover, MACTIDE‐V augmented the number of NK cells, in line with studies reporting that immunosuppressive TAM inhibits NK cells.^[^ ^46^ ^]^ These immunostimulatory aspects of MACTIDE‐V likely account for its anti‐tumoral effect. The anti‐metastatic effect of MACTIDE‐V may be explained by the reduction of “M2‐like” TAM (here CD206^+^MHCII^−^), cells with a major role in breast cancer metastasis.^[^ ^47^ ^]^ A similar upregulation of pro‐inflammatory and anti‐tumoral markers was observed in vitro in BMDM after MACTIDE‐V treatment, further strengthening the idea that this PDC modulates macrophages toward an M1‐like or a mixed M1‐M2 phenotype.

Besides its expression in immunosuppressive M2‐like TAM, CD206 is also expressed by a subset of TAM which participates in antigen uptake and presentation and stimulates anti‐tumoral immunity.^[^ ^48^ ^]^ Moreover, CD206 is expressed by phagocytic macrophages.^[^ ^49^ ^]^ Interestingly, a correlation between the density of CD206^+^ TAM and smaller tumor size and relapse‐free survival has been reported in a cohort of TNBC patients.^[^ ^50^ ^]^ For these reasons, and supported by the findings of our paper, the approach of modulating the phenotype of CD206^+^ TAM, instead of depleting these cells,^[^ ^24^ ^]^ is likely a more effective strategy for breast cancer therapy.

Treatment combining MACTIDE‐V and anti‐PD‐1 did not show synergy. Phagocytic macrophages were previously shown to interfere with T‐cell‐based therapies: Arlauckas et al. found that phagocytic TAM took up the administered anti‐PD‐1 from the surface of the T‐cells and Yamada‐Hunter et al. showed that activating macrophage phagocytosis can lead to TAM phagocytosis of chimeric antigen receptor (CAR)‐T cells.^[^ ^52^ ^]^ In our study, we noticed that PD‐1 was downregulated after MACTIDE‐V treatment. This depletion of PD‐1 from T cells – the intended target of administered anti‐PD‐1–possibly affected the efficacy of anti‐PD‐1 therapy.

These results highlight the challenges in designing TAM‐aimed together with T cell‐aimed therapies and the need for further studies to fully comprehend these complexities and optimize treatment schedules.

In practical terms, however, our results support the safety and consistent anti‐tumor effect of MACTIDE‐V monotherapy. Given the strong anti‐metastatic effect of MACTIDE‐V, one would envision applying MACTIDE‐V as a neo‐adjuvant agent in metastatic breast cancer before resection of the primary tumor, or as an adjuvant agent together with chemotherapy, before or after immunotherapy. Further studies will need to assess MACTIDE‐V in other tumor models, delve into the action mechanism, and explore the oral route of administration.

PDCs to deplete TAM have been reported;^[^ ^53^, ^54^ ^]^ however, the use of PDC using orally available macrophage‐targeting peptides to modulate TAM function into an anti‐tumoral phenotype is one novel aspect of our work.

In conclusion, MACTIDE‐V represents a valuable PDC for reprogramming TAM/monocytes and the MACTIDE peptide represents a potent tool to target CD206^+^ TAM, through oral, intravenous, or intraperitoneal administration.

## 4 Experimental Section

### Peptides and Peptide Conjugates

Peptides were synthesized in the solid phase. FAM and Vert denote carboxyfluorescein and Verteporfin respectively and they were coupled to the N‐terminus of peptides via their carboxylic acid, spaced via an aminohexaonic acid linker (Ahx). Peptides and FAM‐peptide conjugates were purchased from Lifetein LLC, TAG Copenhagen or synthesized at the peptide synthesis core facility of CNB‐CSIC, Madrid, Spain.

Verteporfin‐peptide conjugates were prepared in the Peptide Synthesis Unit (U3) at IQAC‐CSIC (https://www.nanbiosis.es/portfolio/u3‐synthesis‐of‐peptides‐unit/). The peptide moiety was synthesized on a microwave‐assisted peptide synthesizer (Liberty Blue, CEM), using Rink amide Protide resin (0.56 mmol g^−1^, CEM) as a solid support and a Fmoc/tBu strategy. Diisopropylcarbodiimide (DIC) and Oxyme were used as coupling reagents. After completion of the peptide moiety, verteporfin (2 eq.) was manually introduced. Vert‐peptide conjugates were released from the solid support by treatment with TFA: CH_2_Cl_2_:TIS (95:2.5:2.5, v/v/v) for 1.5 h. The solvent was then evaporated under vacuum, and the peptide conjugates were precipitated with cold diethyl ether and centrifugated. Then, the liquid was decanted and the solid dissolved in a mixture of H_2_O:CH_3_CN (1:1, v/v) and lyophilized. To generate the MACTIDE‐V with the disulfide bridge, a 1 mm solution of the linear precursor of MACTIDE‐V in H_2_O:CH_3_CN (1:1, v/v) was prepared and the pH was adjusted to 8 with a solution of 20% NH_4_OH in H_2_O. The evolution of disulfide formation was monitored by HPLC and was completed after 12 h. MACTIDE‐V was purified by semipreparative HPLC with an XBridge Peptide BEH C18 OBD Prep column (130 Å, 5 µm, 19×100 mm), using H_2_O (1% CF_3_COOH) and CH_3_CN (1% CF_3_COOH) as eluents. Final pure peptide conjugates were analyzed and characterized by HPLC and HPLC‐MS (**Table** **1**).

**Table 1:** Peptides and conjugates used were the following.

Notation used	Peptide	Notes
FAM‐MACTIDE	FAM‐Ahx‐G(CTKSIPPIC)SPGAK‐OH	Disulfide: C2‐C10 Ahx: aminohexanoic acid
MACTIDE	G(CTKSIPPIC)SPGAK‐OH	Disulfide: C2‐C10
mUNO	CSPGAK‐OH	
Ctrl1	Ac‐(CESPLLSEC)‐NH2	Disulfide: C1‐C9 Ac: acetylated
Ctrl2	Ac‐(CRGDKGPDC)‐NH2	Disulfide: C1‐C9 Ac: acetylated
CtrlPep‐V	Vert‐Ahx‐AKPCGS‐OH	Ahx: aminohexanoic acid
MACTIDE‐V	Vert‐Ahx‐G(CTKSIPPIC)SPGAK‐OH	Disulfide: C2‐C10 Ahx: aminohexanoic acid

### Molecular Dynamics

MACTIDE was constructed with tLeap from Amber Package,^[^ ^55^ ^]^ modeling interactions using the ff14SB amber forcefield.^[^ ^56^ ^]^ It was solvated with TIP3P water,^[^ ^57^ ^]^ and Cl^−^ ions were added to neutralize the net charge. Three independent molecular dynamics simulations were performed, each 400 ns long. Simulations were conducted using the Amber18 Package with the following protocol: first, a minimization was performed to relax clashes with the steepest descent method combined with the conjugate gradient. Next, temperature and pressure were included with short simulations using NVT and NPT ensembles and once the systems were equilibrated the production runs were started. The time step used was 2 fs. Electrostatic interactions were treated using particle‐mesh Ewald (PME)^[^ ^58^ ^]^ with a cut‐off of 10 Å. Temperature was regulated using Langevin dynamics^[^ ^59^ ^]^ with a collision frequency of 2 ps^−1^. Simulation trajectories were saved every 10 ps. Representative conformations were extracted from the trajectories by performing a clustering analysis, using a hierarchical agglomerative approach. MACTIDE was solvated with water and two independent molecular dynamics simulations were performed, each 400 ns long, to construct an ensemble of configurations.

### Peptide Docking Analysis

Docking was conducted using HPEPDOCK,^[^ ^15^ ^]^ which involves global sampling of binding orientations along the receptor surface. This algorithm accounted for peptide flexibility by generating an ensemble of conformations, which were then globally docked against the entire protein. To address receptor flexibility, an ensemble of receptor conformations from a previously generated trajectory was used (3). These conformations were clustered based on an RMSD criterion. The five most populated receptor configurations were selected and used as coordinates for the receptor in each docking calculation.

### Peptide Stability in Tumor Lysate

For peptide stability measurements, 200 µL of freshly prepared tumor lysate was mixed with 50 µL of PBS (as control), 30 µm of FAM‐mUNO, or 30 µm of FAM‐MACTIDE and incubated at 37 °C. 40 µL aliquots were taken at 0, 10, 30, 60, 180 and 1440 min. 80 µL of methanol was added to each aliquot and it was immediately stored at −80 °C until analysis later on the same or the following day. For analysis, the samples were centrifuged at 21 000 × g for 10 min at +4 °C. The supernatant was transferred into liquid chromatography (Agilent 1200 series) autosampler and maintained at +4 °C. 10 µL was injected and separation was achieved with a C18 column (Kinetex 2.6 µm EVO C18 100×4.6 mm, Phenomenex). The chromatography gradient started with 5 min 5% acetonitrile in water, followed by a linear increase to 100% acetonitrile in 20 min and finally 20 min isocratic flow of 100% acetonitrile. Both eluents contained 0.2% formic acid. An enhanced resolution scan (Qtrap 4500, Sciex) for peptides with 1, 2, or 3 charges was done. Additionally, all m/z values in the 50–2000 range were scanned for degradation product search. Potential product signals were subjected to fragmentation analysis. Statistics were done using GraphPad Prism 5.0.

### Peptide Binding Studies using Quartz Crystal Microbalance

The Quartz Crystal Microbalance (QCM) used was a QCM200 system from Stanford Research Systems (Sunnyvale, CA, USA). The quartz crystals used have a 5 MHz resonant frequency and were deposited with a layer of Cr/Au (Cat # O100RX1, p/n 6–613, Stanford Research Systems). The crystal was mounted on the cell, and washed with isopropanol, ethanol, and mQ water. Then, it was incubated with 20 mm Mercapto‐propane sulfonate (MPS, Cat #251682, Sigma–Aldrich) in 10 mm H_2_SO_4_ for 30 min, washed with mQ and then incubated with a 10 µm (in monomer) of Polyallylamine hydrochloride, Mw: 50000, PAH, Cat # 283223, Sigma–Aldrich) in mQ at pH 8.5 during 10 min and later washed with mQ. Then, a baseline with 500 µL of mQ at pH 8 was recorded, the measurement was paused and then 5 µL of a 1 mg mL^−1^ solution in PBS was added (human recombinant CD206, Cat # 2534‐MR‐050/CF from R&D systems, reconstituted with 50 µL of mQ); final concentration of CD206 in the cell: 0.01 mg mL^−1^. Of note, the isoelectric point of CD206 is 6.3. For the PAH‐BSA multilayer, Bovine Serum Albumin (BSA, Cat # A7906, Sigma–Aldrich) was deposited, after measuring a baseline in mQ, at a concentration of 0.01 mg mL^−1^ in mQ for 10 min, and then washed with mQ. For peptides, 500 µL of PBS was placed in the cell and the baseline was recorded, the measurement was then paused and 50 µL of a 100 µm solution of peptides in PBS was gently deposited on the cell and the measurement was resumed. Then, the measurement was paused, the solution was removed and replaced with 500 µL of new PBS, and the measurement resumed. The association and dissociation curves were fitted using TraceDrawer software (Ridgeview Instruments AB), to obtain the association constant k_a_, the dissociation constant k_d,_ and the affinity constant K_D_.

### Cell Culture and Experimental Animals

4T1 and 4T1.2 cells were both purchased from ATCC. 4T1 cells were grown in RPMI1640 media (Gibco, catalog no. 72400‐021) supplemented with 10% v/v fetal bovine serum (FBS, Capricorn Scientific, catalog no. FBS‐11A) and 100 IU mL^−1^ penicillin/streptomycin (Pen/Strep, Capricorn Scientific, catalog no. PS‐B) at +37 °C in the presence of 5% CO_2_. 4T1.2 cells were grown in AlphaMEM (Gibco™, catalog no. 12571063) supplemented with 10% v/v FBS and 100 IU mL^−1^ Pen/Strep at +37 °C in the presence of 5% CO_2_.

All animal experiments were performed on 8–12‐week‐old female Balb/c mice and were approved by the Estonian Ministry of Agriculture (project no. 197). All methods were performed in accordance with existing guidelines and regulations.

### In Vivo Biodistribution Studies

Orthotopic tumors were induced by injecting subcutaneously 1×10^6^ 4T1 cells in 50 µL of PBS (Lonza, catalog no. 17–512F) into the 4th mammary fat pad of 8–12‐week‐old female Balb/c mice. When tumors reached ≈100 mm^3^, 30 nmol of FAM‐MACTIDE or FAM‐mUNO was injected i.p., i.v. or through oral gavage and circulated for 24 h. Then, mice were sacrificed by anesthetic overdose and cervical dislocation, and organs and tumors were collected and fixed in cold 4% paraformaldehyde (PFA) in PBS at +4 °C overnight followed by washing in PBS at RT for 1 h after which 15% w/v sucrose was added for 24 h. Finally, 30% sucrose was added overnight, cryoprotected tissues were frozen in Optimal Cutting Temperature (OCT; Leica, catalog no. 14020108926) media and stored at −80 °C for long term or at −20 °C for short term. Blocks were cryosectioned at 10 µm thickness on Superfrost+ slides (ThermoFisher Scientific, catalog no. J1800AMNZ) and stored at −20 °C or used instantly. Immunofluorescence staining was performed as described previously.^[^ ^5^ ^]^ CD206 was detected using rat anti‐mouse CD206 (dilution 1/200, Bio‐Rad, catalog no. MCA2235GA) and Alexa Fluor 647 goat anti‐rat antibody (dilution 1/300). FAM was detected using rabbit anti‐ FAM (dilution 1/100, Thermo Fisher Scientific, catalog no. A889) and Alexa Fluor 546 goat anti‐rabbit antibody (dilution 1/200). Slides were counterstained using 4,6‐diamidino‐2‐phenylindole (DAPI, 5 µg mL^−1^ in PBS, Sigma–Aldrich, catalog no. D9542‐5MG). Slides were mounted using a mounting media (Fluoromount‐G Electron Microscopy Sciences, catalog no. 17984‐25) and imaged using a Zeiss confocal microscope (Zeiss LSM‐710) and 20 x objective. The colocalization analysis was performed using the “Coloc2” plugin in the Fiji program using Mandler's tM2 index. Values were obtained from at least three individual images per mouse per group and their average values were plotted. The FAM mean signal per CD206^+^ cell analysis was measured using Fiji, taking the mean FAM signal, and dividing it by the number of CD206^+^ cells. Average values were obtained from four images per mouse for *n* = 3 mice.

### In Vitro Photodynamic Therapy

Human peripheral mononuclear cells (PBMCs) were purified from human blood buffy coats following the protocol described previously.^[^ ^24^ ^]^ Briefly, Ficoll Paque Plus (GE Healthcare, catalog no. 17‐1440‐02) reagent and CD14^+^ microbeads (MACS Miltenyi Biotec, catalog no. 130‐050‐201), seeded 1.2×10^5^ cells in 50 µL of RPMI1640 media on FBS‐coated 96‐well plate was used. For optimal cell attachment and polarization, a macrophage colony‐stimulating factor (M‐CSF, 50 ng mL^−1^, BioLegend, catalog no. 574802) was added. Then, to obtain M2 resembling phenotype, monocytes were stimulated with IL‐4 (50 ng mL^−1^, BioLegend, catalog no. 574002). 50 µL of media containing M‐CSF and IL4 was replenished every other day for 7 days. To obtain M1 resembling phenotype, monocytes were stimulated with M‐CSF for 6 days (50 ng mL^−1^), 50 µL replenished every other day after which lipopolysaccharide (LPS, 100 ng mL^−1^, Sigma–Aldrich, catalog no. L4391) and IFNγ (20 ng mL^−1^, BioLegend, catalog no. 570202) were added and incubated overnight. All incubations were done at +37 °C. On day 7, 30 µm of MACTIDE‐V, mUNO‐V, CtrlPep‐V, DOX, or PBS was added, and cells were incubated for 60 min at + 37 °C after which cells were washed with media and 100 µL of new RPMI without phenol red (Gibco, catalog no. 11835030) was added. *n* = 3 wells/group from *n* = 6 donors. Cells were then irradiated using a NIR laser source for PDT from Modulight Inc (ML6500, 2W, 689 nm) and an optical fiber with frontal diffuser (SMA905, Modulight), dose 10 J cm^−2^ and spot size 0.5 cm. As Verteporfin is light‐sensitive, everything was performed in the dark. To keep conditions the same, the plate that did not receive irradiation was also kept open for the same about of time. After irradiation, cells were incubated at +37 °C for 48 h. To analyze cell death, 10 µL of 4,5‐Dimethylthiazol‐2‐yl)‐2,5‐Diphenyltetrazolium Bromide (MTT, 5 mg mL^−1^) in PBS was added to the cells and incubated at +37 °C up to 90 min. Crystal formation was monitored every 20–30 min to not oversaturate the absorbance values. Then, the media was removed carefully and 100 µL of DMSOl was added to each well, and the plate was shaken until all crystals were dissolved. Absorbance was read at 570 nm using a plate reader (Tecan Sunrise) and a corresponding program (Magellan 7).

### In Vivo Photodynamic Therapy in Orthotopic 4T1

Orthotopic tumors were induced by injecting 5×10^4^ 4T1 cells in 50 µL of PBS subcutaneously into the 4th mammary fat pad of 8–12‐week‐old female Balb/c mice. When tumors reached ≈40 mm^3^, mice were sorted into groups: MACTIDE‐V (+), MACTIDE‐V (‐), CtrlPep‐V (+), CtrlPep‐V (‐), MACTIDE (‐), and PBS (+). Tumors were measured using a digital caliper (Mitutoyo) and volume was calculated using (W^2^ × L)/2 formula, where W is the width of a tumor and L is the length. Each group had six mice. The first intraperitoneal injection (30 nmol) was carried out on day 9 post‐tumor induction. 4 h post‐injection, the tumor area was shaved to lessen laser scattering and tumors were irradiated with 100 J cm^−2^ using the NIR laser source for PDT described above. Each tumor was measured, and the spot size was adjusted every time, keeping the radiation dose always constant. Irradiation of the tumors was done in complete darkness under anesthesia. Mice recovered on a heating pad and eye drops were applied to avoid eye dryness. All mice, whether irradiated or not, were anesthetized to keep the handling conditions the same. Mouse bodyweights and tumor volumes were monitored every other day. The sacrifice of mice began on day 23 based on their tumor sizes (over 1500 mm^3^) or cachexia. The final mice were sacrificed on day 36 post‐tumor induction. Tumor volume curves are shown until day 23 when mice elimination began, and the sample number became too small for statistical comparison. Survival was analyzed using GraphPad Prism to plot Kaplan–Meier survival curves and perform the Mantel–Cox test for statistical analysis. Tumors taken on similar days were analyzed using mIHC.

### Multiplex Immunohistochemistry (mIHC)

The staining of 4 µm‐thick formalin‐fixed paraffin‐embedded (FFPE) tissue sections for mIHC analysis was carried out using the tyramide signal amplification‐based Opal method (Akoya Biosciences) on a Leica BOND RX automated immunostainer (Leica Microsystems). For each staining cycle, FFPE slides were deparaffinized and subjected to heat‐induced epitope retrieval at 97 or 100 °C using BOND epitope retrieval solutions ER1 or ER2 (Leica Biosystems). The tissue sections were incubated with a blocking solution for 10 min, then incubated for 30 min with primary antibodies listed in the table below (**Table** **2**).

**Table 2:** Antibodies used for multiplex immunohistochemistry.

Antigen	Clone	Reference	Dilution	Incubation time	Secondary antibody	OPAL
CD4	4SM95	Invitrogen 14‐9766‐82	1:150	30′	anti‐rat	480
CD8	4SM15	Invitrogen 14‐0808‐82	1:150	30′	anti‐rat	620
Ly6G	EPR 22909‐135	Abcam ab238132	1:500	30′	anti‐rabbit	570
Fibronectin	polyclonal	Abcam ab2413	1:250	30′	anti‐rabbit	690
FOXP3	221D	Abcam ab253297	1:200	30′	anti‐mouse	650
Cytokeratin 8	EP1628Y	Abcam ab53280	1:200	30′	anti‐rabbit	780

The slides were then incubated for 10 min with the horseradish peroxidase‐conjugated secondary antibodies Opal polymer HRP mouse‐rabbit (Akoya Biosciences), ImmPRESS HRP Goat Anti‐Rat IgG HRP‐polymer or ImmPRESS HRP Goat Anti‐Rabbit IgG HRP‐polymer (Vector Laboratories). After washing, the slides were incubated for 10 min with the tyramide signal amplification‐conjugated fluorophores Opal‐480, 570, 620, 650, 690, and 780 (Akoya Biosciences) and with the spectral DAPI (Akoya Biosciences) as a nuclear counterstain. After washing, the slides were mounted using the ProLong diamond antifade mountant (Invitrogen, ThermoFisher Scientific) and imaged using a Mantra2 quantitative pathology workstation (Akoya Biosciences) at X20 magnification. At least 20 fields were acquired for each slide. The spectral unmixing of the images was performed with InForm 2.6 Image Analysis Software (Akoya Biosciences) and the analysis of cell density with QuPath v0.5.1. Fibronectin and Ly6G staining (not shown) were used to better identify non‐necrotic tumor regions.

### RNA Extraction, Retro Transcription, and Real‐Time PCR

For RNA extraction, BMDM were detached, pelleted, and resuspended in 1 mL of TRIzol reagent (Cat# 15596018, Invitrogen, ThermoFisher Scientific) and stored at −80 °C until the RNA extraction. Total RNA was extracted following TRIzol manufacturer's protocol and RNA was quantified using NanoDrop One (Thermofisher Scientific). 1000 ng of RNA was used for cDNA retrotranscription using the High‐capacity RNA‐to‐cDNA™ Kit (Cat# 4387406, Applied Biosystems, Thermofisher Scientific) following the manufacturer's instructions. 20 ng of cDNA per well were amplified in 20 µL using the TaqMan Universal PCR Master Mix (Cat# 4364340) and TaqMan assay primers and probes (Cat# 4448892 or 4453320, Applied Biosystems, Thermofisher Scientific). The reaction was performed in a MicroAmp Optical 96‐well reaction plate (Cat# N8010560, Applied Biosystems, Thermofisher Scientific) and analyzed on a QuantStudio 7 Pro Real‐Time PCR System. All the samples were amplified in duplicates and data were analyzed using the ΔΔCt method using *Gapdh* as a housekeeping gene (**Table** **3**).

**Table 3:** Taqman Gene Expression Assays used for Real‐ Time PCR.

Gene	Taqman assay ID
*Tnfa*	Mm00443258_m1
*Il1b*	Mm00434228_m1
*Il12*	Mm00434174_m1
*Il10*	Mm00439614_m1
*Ctgf*	Mm01192933_g1
*Cyrg1*	Mm00487498_m1
*Chil3*	Mm00657889_mH
*Nos2*	Mm00440485_m1
*Cxcl9*	Mm00434946_m1
*Itgb5*	Mm00439825_m1
*Cdca7 *	Mm00788027_s1
*Cyp1b1*	Mm00487229_m1
*F3*	Mm00438855_m1
*Il‐6*	Mm00446190_m1
*Gapdh*	Mm99999915_g1

### Mouse BMDM Differentiation and Treatment with Conjugates

Bone marrow cells were isolated from 8 to 12‐week‐old female Balb/c mice by flushing the femur and tibia bones with media using a 25G needle. Red blood cells were lysed (RBC lysis buffer for mouse, ThermoScientific, cat # J62150.AK), and cell suspensions were washed, resuspended in a complete media, and filtered with a 100 µm cell strainer. Macrophages were obtained by culturing the bone marrow cells for 5 days at a density of 2×10^5^ cells cm^−2^ in RPMI1640 (Gibco, cat # 11875‐093) supplemented with 10% (v/v) FBS and 1% penicillin/streptomycin, and 100 ng mL^−1^ M‐CSF (BioLegend, cat # 576406) at 37 °C, 5% CO_2_. Culture media was refreshed every 2–3 days by substituting half of the media with a fresh one containing M‐CSF. On day 5, the cell culture media was removed and replenished with an equal volume of media containing the peptide conjugates at the final concentration of 10 µm. After an incubation of 4 h at 37 °C, the peptide conjugates were removed by a washout and BMDM were further incubated for 48 h at 37 °C with fresh media.

### YAP Immunofluorescence

For YAP immunostaining, cells were cultured on the Ibidi plate (Ibidi, cat# 80821) previously coated with FBS and treated with conjugates as previously described. 3 h after peptide conjugates were removed, the media was also removed, and cells were washed with PBS and fixed with PFA (ThermoScientific, Cat # J61899.AP). Fixed cells were then washed with PBS, permeabilized with PBS‐0.2% Triton‐X100 (v/v), and washed with PBS 0.05% Tween‐20 (PBS‐T) (v/v), before being incubated with blocking buffer (5% BSA (w/v), 5% FBS (v/v) in PBS‐T). After blocking, cells were incubated overnight at +4 °C with rabbit anti‐YAP1 (LS Bio, cat # LS‐C331201‐20) at a dilution of 1/300 in diluted blocking buffer (dBB, 1/5 dilution of blocking buffer in PBS‐T). The following day, cells were washed with PBS‐T and incubated for 35 min at RT with secondary antibody A647 goat anti‐rabbit (Invitrogen, Cat # A21245) at a dilution of 1/500 in dBB. Secondary antibody was later washed with PBS‐T and PBS and after that, cells were incubated for 5 min at RT with DAPI (5 µg mL^−1^) followed by washing steps with PBS‐T and PBS. Cells were imaged in PBS using a Zeiss LSM780 confocal microscope using the 40X objective.

### Phagocytosis Assays

For the *E. coli* phagocytosis assay, cells were cultured in a 96‐well plate and treated with conjugates as previously described (for 4 h at a 10 µm concentration at 37 °C, then peptide conjugates were removed, washed with media and fresh media was added). The phagocytosis assay was performed at 48 h after treatment removal by removing the media, incubating fluorescent E. coli BioParticles for 2 h, followed by a 1 min incubation with trypan blue (Vybrant Phagocytosis Assay Kit, Invitrogen, Cat# V6694). Plates were read at 480 nm excitation and 520 nm emission using a BioTek Synergy H1 plate reader.

For the live 4T1‐GFP phagocytosis assay, 4T1‐GFP cells were cultured in RPMI1640 supplemented with 10% (v/v) FBS and 1% penicillin/streptomycin at 37 °C, 5% CO_2_. BMDM were seeded on Ibidi plates (Ibidi, cat# 80821) and at day 5 were treated with MACTIDE and conjugates as previously described (10 µm during 4h, followed by washing with media). Then, 48h later, the BMDM were incubated for 2 h with 2×10^5^ 4T1‐GFP cells (the area of the wells is 1 cm^2^). Then, the supernatant was removed, and the wells were washed with media and then with PBS. Then, the cells were fixed with ice‐cold acetone for 10 min. Fixed cells were then washed with PBS, permeabilized with PBS‐0.2% Triton for 10 min and washed with PBS‐T (PBS+0.05% Tween‐20 (v/v)), and then incubated with blocking buffer (5% BSA (w/v), 5% FBS (v/v) in PBS‐T) for 1 h at room temperature. After blocking, the cells were incubated with Alexa Fluor 647 anti‐GFP (BioLegend, cat# 338005) or its isotype control (BioLegend, cat# 400155) at a final concentration of 2 µg mL^−1^ in diluted blocking buffer (1/5 dilution of blocking buffer in PBS‐T) overnight at +4 °C. The following day, cells were washed with PBS‐T and PBS and after that, they were incubated for 5 min at room temperature with DAPI (5 µg mL^−1^) followed by washing steps with PBS‐T and PBS. Cells were imaged in PBS using a Zeiss LSM780 confocal microscope and a 40X objective. The mean GFP signal intensity was quantified from the images using ImageJ and normalized to the amount of DAPI^+^ nuclei.

### In Vitro Cell Viability in BMDM

Day 5 BMDM in 96‐well plates were treated with 10 µm MACTIDE, conjugates, or Verteporfin (dissolved in 0.1% DMSO) for 4 h, followed by washing with media and placing fresh media. Then, 48h later, 10 µL of MTT (5 mg mL^−1^) in PBS was added to the cells and incubated at 37 °C for 2.5h. Then, the media was removed carefully and 100 µL of DMSO was added to each well, and the plate was shaken until all crystals were dissolved. Absorbance was read at 570 nm using a plate reader.

### MACTIDE‐V Monotherapy in Orthotopic 4T1.2

5×10^5^ 4T1.2 cells in 50 µL of PBS was injected subcutaneously to 4th mammary fat pad. On day 7, mice were sorted into groups based on tumor volume, ≈55—60 mm^3^ calculated based on the formula shown above. Mice were treated every other day with 500 µL of MACTIDE‐V, CtrlPep‐V (30 nmol), and PBS, 9 injections in total, *n* = 10 mice/group. The cumulative Verteporfin dose was 9 mg kg^−1^. The last injection was on day 23. Four mice from each group were sacrificed on day 25 through anesthetic overdose and their tumors were analyzed by flow cytometry (FC) and mIHC. The rest of the mice were left to continue in the study with the initial intention of performing a survival study, however, all of these mice were sacrificed within 5 days based on their tumor size (above 1500 mm^3^) or cachexia.

### Flow Cytometry

For FC, mice were not perfused, the tumors were cut into small pieces, digested using 10 mL of collagenase IV (200 U mL^−1^, Gibco, catalog no.17104019), dispase (0.6 U mL^−1^, Gibco, catalog no. 17105‐041) and DNase I (15 U mL^−1^, AppliChem, catalog no. A3778) mixture on a rotating platform for up to 60 min at 37 °C, pipetting every 10 min. Red blood cells were lysed using 3 mL of ammonium‐chloride‐potassium lysis buffer. After that, cells were centrifuged (350×g, 7 min, +4 °C), filtered (100 µm cell strainer, Falcon, catalog no. 352360), and counted using the bright‐field mode of LUNA Automated Cell counter (Logos Biosystems). Cells were seeded at a concentration of 5×10^6^ cells/100 µL of running buffer (RB) (1L of RB: 4 mL 0.5m EDTA + 100 mL FBS + the rest PBS) on the 96‐well conical bottom plate and incubated for 15 min in dark at RT in 50 µL of Zombie NIR Fixable Viability Kit (BioLegend). After that, 30 µL of blocking antibody (TruStain FcX, Biolegend) was added and incubated for 10 min in the dark at +4 °C. Then, to stain macrophages, 20 µL of antibody mixture was added, and incubated for 25 min in the dark at +4 °C after which 50 µL of RB was added, centrifuged, washed two times with 150 µL of RB, and taken up in 150 µL of RB. For intracellular staining, cells were fixed and permeabilized using the eBioscience Foxp3/Transcription Factor Staining Buffer Set (Thermofisher). Cells were then stained with 100 µL of antibody mixture and incubated for 40 min in the dark at RT. Cells were centrifuged, washed twice with 150 µL of RB, and resuspended in 150 µL of RB. Samples were analyzed using the SONY ID7000 spectral flow cytometer and the accompanying software (**Table** **4**).

**Table 4:** Antibodies used for in vivo flow cytometry.

Antigen	Fluorophore	Clone	Catalog number	Company
CD86	BV421	GL‐1	105032	Biolegend
PD‐1	BV510	29F.1A12	135241	Biolegend
LY6C	SBV570	ER‐MP20	MCA2389SBV570	Bio‐Rad
CD11b	BV750	M1/70	101267	Biolegend
CD3	FITC	145‐2C11	100306	Biolegend
MHC II	NovaFluorBlue610	M5/114.15.2	# M024T02B06	Thermofisher
F4/80	SBB765	Cl:A3‐1	MCA497SBB765	Bio‐Rad
CD45	SBB810	YW62.3	MCA1031SBB810	Bio‐Rad
CD206	PE‐Fire700	C068C2	141742	Biolegend
PDL1	NovaFluorRed700	MIH5	M036T03R03	Thermofisher
CD11b	APC‐Cy7	N418	117324	Biolegend
B220	APC‐Fire810	RA3‐6B2	103278	Biolegend
GRZB	BV421	QA18A28	396414	Biolegend
CD49	PacBlue	DX5	108918	Biolegend
CD4	BV570	RM4‐5	100542	Biolegend
CD27	BV605	LG 3A10	124249	Biolegend
CD8	BV650	53‐6.7	100742	Biolegend
CD44	BV711	IM7	103057	Biolegend
FOXP3	AF488	150D	320012	Biolegend
CD3	SparkBlue‐574	17A2	100276	Biolegend
CD19	SBB765	6D5	MCA1439SBB765	Bio‐Rad
IL7RA	PE‐Cy7	A7R34	135014	Biolegend
CD25	APC	PC61	102012	Biolegend
KI67	AF700	16A8	652420	Biolegend
CD62L	APC‐Cy7	MEL‐14	104428	Biolegend

For FC of mouse BMDM, macrophages were detached at day 7, 48 h after treatment with either PBS or PDCs, with cold PBS 2 mm EDTA. Cells were pelleted, washed, and stained in PBS for 20 min at 4 °C with the LIVE/DEAD Fixable Scarlet (723) Viability Kit (Cat# L34986, Invitrogen, ThermoFisher Scientific) and the antibodies listed below (**Table** **5**).

**Table 5:** Antibodies used for flow cytometry of BMDM.

Antigen	Fluorophore	Clone	Catalog number	Company
CD11b	PE‐Cy7	M1/70	552850	BD
F4/80	FITC	Cl:A3‐1	MCA497F	Bio‐Rad
CD206	AF647	MR5D3	565250	BD
I‐A/I‐E (MHC II)	PERCPCy5.5	M5/114.15.2	562363	BD
CD274 (PD‐L1)	SB645	MIH5	64‐5982‐82	Invitrogen
CD80	PE	16‐10A1	12‐0801‐81	eBioscience

### Metastasis Assessment

To determine the pulmonary metastatic area, lungs from MACTIDE‐V monotherapy were stained for hematoxylin and eosin according to the following protocol. Unfixed slides (10 µm in thickness) were taken out of the −20 °C freezer 10 min before starting the staining. First, slides were fixed for 2 min in ice‐cold methanol, then incubated for 3 min in hematoxylin solution after which slides were washed for 5 min with running tap water. Then, slides were incubated for 3 min in eosin solution and later washed for 5 min in running water. For rehydration, slides were placed two times into 100% ethanol for 1 min and then for clearance two times into RotiClear (Roth, catalog no. A538.5) for 2 min after which slides were mounted using Eukitt quick hardening mounting medium (Merck, catalog no. 03989). Slides were then scanned using Leica DM6 B microscope and Leica Aperio Versa 8 slide scanner with 20x zoom. Images were analyzed using the QuPath program to determine the pulmonary tumor area coverage by dividing the tumor area per whole lung area and multiplying by 100.

### MACTIDE‐V + Anti‐PD1 Combination Therapy in Orthotopic 4T1.2

5×10^5^ 4T1.2 cells in 50 µL of PBS were injected subcutaneously into 4th mammary fat pad. On day 8, mice were sorted into groups based on tumor volume (≈40 mm^3^): PBS, anti‐PD‐1, MACTIDE‐V, MACTIDE‐V+anti‐PD‐1. Mice were treated i.p. every other day with 500 µL of MACTIDE‐V (30 nmol, 1mg kg^−1^ Verteporfin) or PBS (10 injections in total). On day 18 post‐injection, MACTIDE‐V+anti‐PD‐1 and anti‐PD‐1 groups received recombiMAb anti‐mouse PD‐1 (Bioxcell, Catalog #CP151), a mouse IgG2a monoclonal antibody with the D265A mutation in the Fc fragment which renders it unable to bind to endogenous Fcγ receptors. Mice received three intraperitoneal injections of 200 µg of anti‐PD‐1 dissolved in 500 µL of PBS. The groups that did not receive anti‐PD1 were injected with 500 µL of PBS i.p. The last injection was on day 27, all mice were sacrificed on day 29 through an anesthetic overdose. Five tumors per group were analyzed by FC as described above. TAM/monocytes were defined as Ly6G^−^CD11b^+^/F4/80^+^ population from the CD45^+^ population.

The lungs of all mice were fixed in formalin and embedded in paraffin, and later sectioned and stained with H&E by the histology facility of Vall D´Hebron Research Institute (Barcelona, Spain). Five 10 µm‐sections spaced apart 1 mm were taken from the lungs of each mouse (*n* = 7 mice per group). Then, the slides were scanned, and the metastatic area divided by the total lung area was calculated for each mouse.

## Conflict of Interest

The authors declare no conflict of interest.

## Author Contributions

A.L., E.P. contributed equally to this work. A.L. performed in vitro and in vivo experiments, designed experiments, wrote the manuscript, and provided analysis and discussion. E.P. performed in vitro experiments with BMDM, designed experiments, wrote the manuscript, and provided discussion and analysis. U.H. performed flow cytometry studies from in vivo experiments, designed experiments, edited the manuscript, and provided analysis and discussions. E.K.A. performed modeling studies. M.C.A. and L.M. designed, analyzed, and performed in vitro experiments with BMDM. K.K. performed LC‐MS studies. M.L, M.R, G.A. provided peptide and peptide‐conjugate synthesis. I.M. and P.P. provided funding and discussion. T.T. provided funding and discussion and edited the manuscript. P.S. performed binding and homing studies, designed experiments, provided supervision, provided funding, and wrote the manuscript.

## Supporting information



Supporting Information

## Data Availability

All data needed to evaluate the conclusions on the article are presented in the article and/or the Supplementary Data. Additional data related to the findings of this study are available from the corresponding author.
