# Supplementary material for: Peptide‐Drug Conjugate for Therapeutic Reprogramming of Tumor‐Associated Macrophages in Breast Cancer
Source: Adv Sci (Weinh). 2025 Jan 22;12(10):2410288. doi: 10.1002/advs.202410288 (PMC11904948; doi:10.1002/advs.202410288)
Supplement: Supplementary file 1 — Supporting Information [file ADVS-12-2410288-s001.docx]

**SUPPORTING INFORMATION**

Peptide-Drug Conjugate for Therapeutic Reprogramming of Tumor-Associated Macrophages in Breast Cancer

Anni Lepland^1 ‡^, Elisa Peranzoni^‡ 2^, Uku Haljasorg^3^, Eliana K. Asciutto^5^, Maria Crespí Amer^4^, Lorenzo Modesti^2^, Kalle Kilk^6^, Manuel Lombardia^7^, Gerardo Acosta^4,9^, Miriam Royo^4,9^, Pärt Peterson^3^, Ilaria Marigo^8,2^, Tambet Teesalu^1^, Pablo Scodeller*^1,4^

^1^ *Institute of Biomedicine and Translational Medicine, University of Tartu, Ravila 14B, Tartu 50411, Estonia.*

*^2^ Immunology and Molecular Oncology Diagnostics, Veneto Institute of Oncology IOV – IRCCS, 35128 Padua, Italy.*

*^3^ Molecular Pathology Research Group, Institute of Biomedicine and Translational Medicine, University of Tartu, Tartu, Estonia.*

*^4^ Institute for Advanced Chemistry of Catalonia, IQAC-CSIC, Jordi Girona 18-26, Barcelona 08034.*

^5^ *Instituto de Ciencias Físicas, Universidad Nacional de San Martin (UNSAM) and CONICET, Campus Migueletes, 25 de Mayo y Francia, Buenos Aires CP 1650, Argentina.*

^6^ *Department of biochemistry, Institute of Biomedicine and Translational Medicine University of Tartu, Ravila 19, Tartu 50411, Estonia.*

*^7^ Proteomics facility, Centro Nacional de Biotecnologia, CNB-CSIC, Calle Darwin 3, Madrid.*

*^8^ Department of Surgery, Oncology and Gastroenterology (DISCOG), University of Padova, 35128 Padova, Italy.*

*^9^ CIBER-BBN, Networking Centre on Bioengineering, Biomaterials and Nanomedicine, IQAC-CSIC, 08034 Barcelona, Spain.*

^‡^ *Equal contribution*

**Corresponding author: pablo.scodeller@iqac.csic.es*


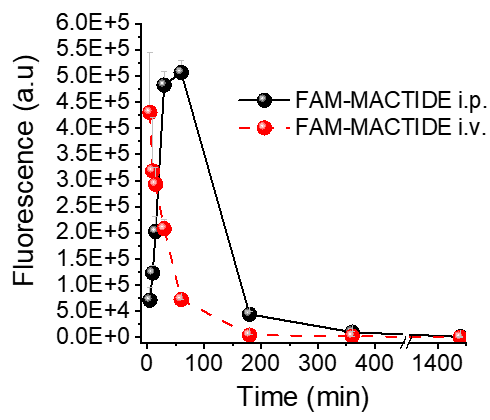


**Figure S1.**: Pharmacokinetics of i.p. and i.v. administered FAM-MACTIDE (30 nmoles) analyzed by detecting plasma fluorescence in the FAM channel at different timepoints (n=3).


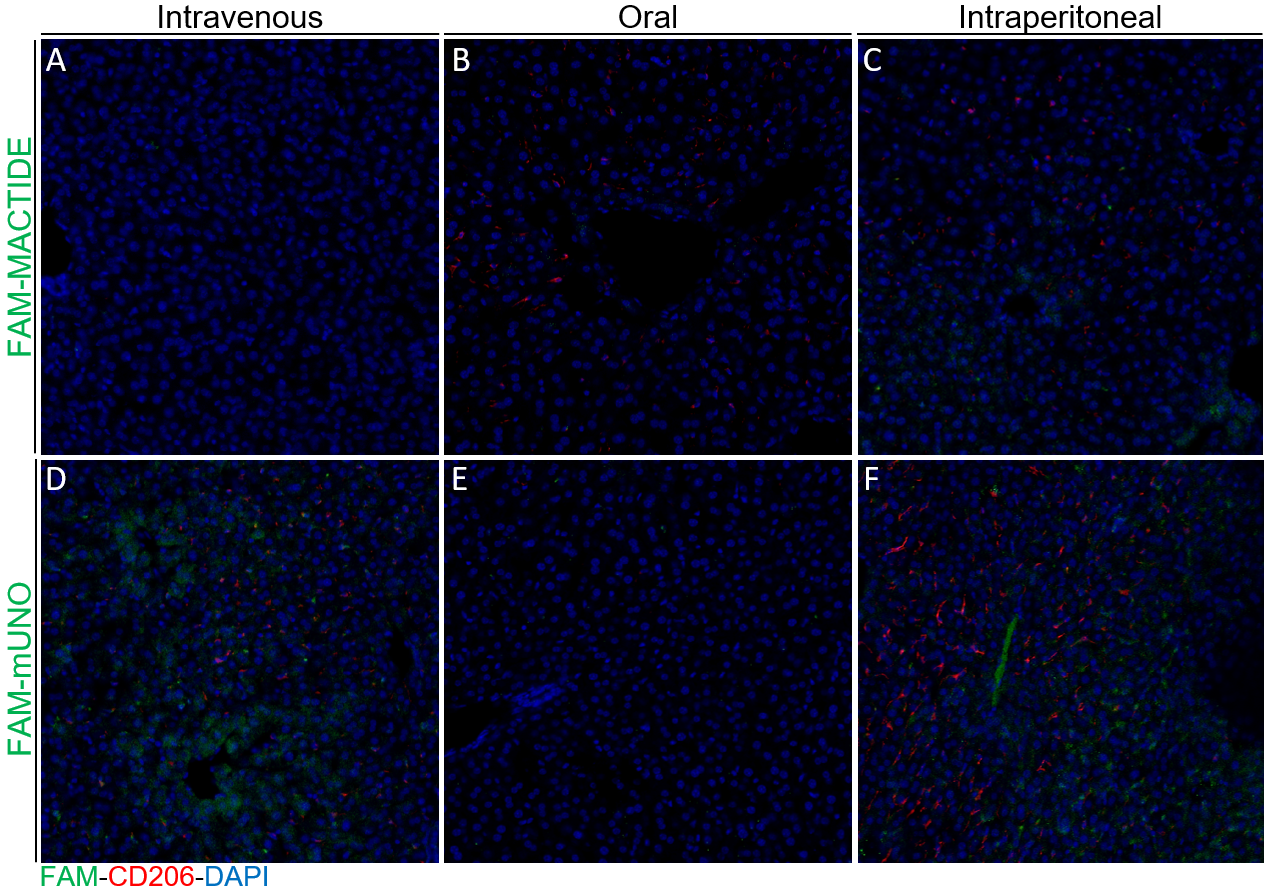


**Figure S2**. Intravenously, intraperitoneally and orally administered FAM-MACTIDE or FAM-mUNO does not accumulate in the liver of breast cancer in mice. 30 nmol of FAM-MACTIDE or FAM-mUNO were administered and left to circulate for 24 h. At 24 h, the mice were sacrificed, and the organs were collected, fixed, cryoprotected, sectioned, and immunostained for FAM (shown in green) and CD206 (shown in red). Representative images from n=3 mice are shown.


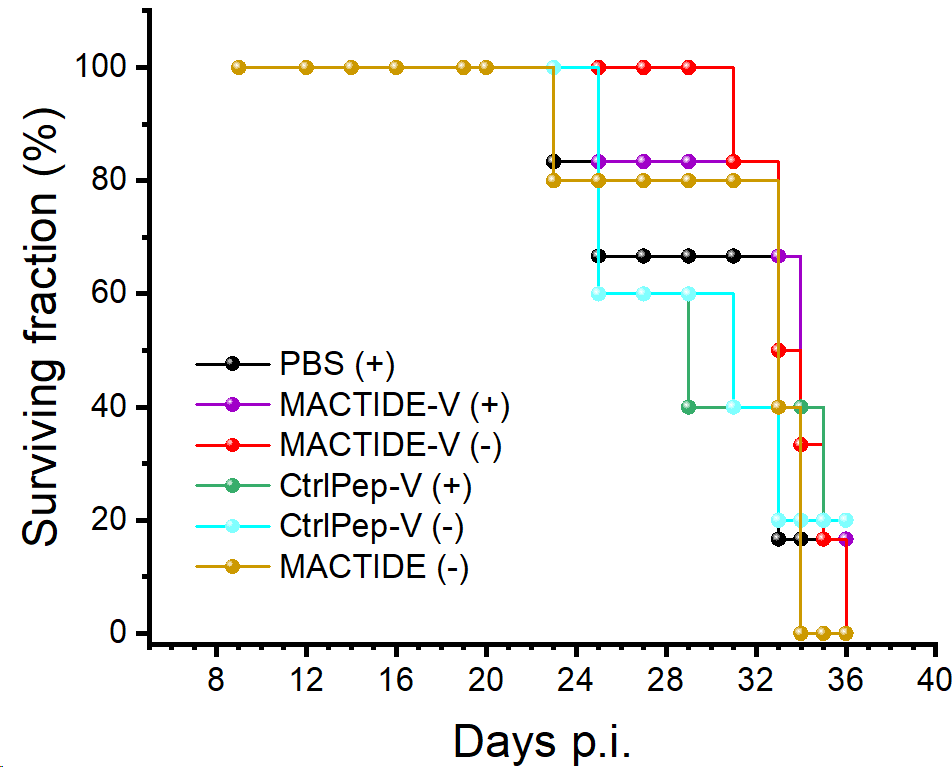


**Figure S3**. Survival for treatment study of figure 5.


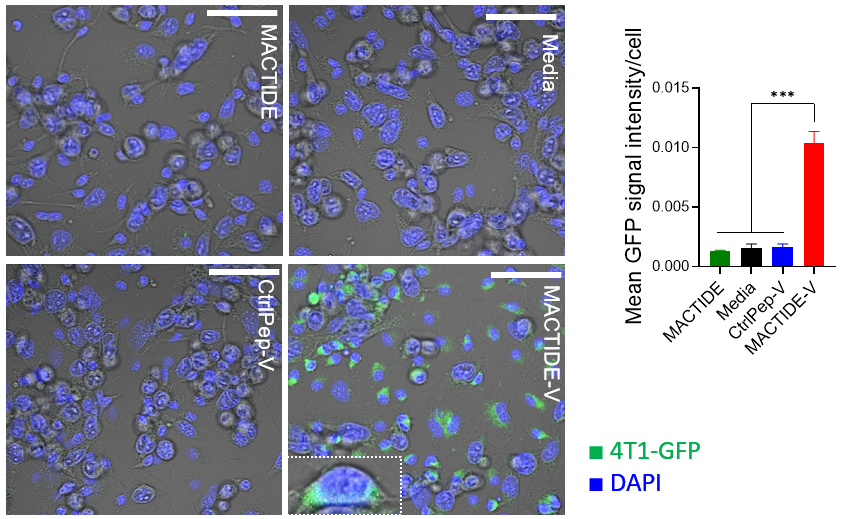


**Figure S4. Phagocytosis of live cancer cells**. BMBM from Balb/c mice were cultured on Ibidi plates (1cm^2^ surface area) treated with 10μM during 4h, subsequently washed, placed in new media and cultured for additional 48 h. Then, 200.000 4T1-GFP cells were added to the wells, and incubated at 37°C for 2 h. After this step, the wells were washed, fixed with ice-cold acetone, permeabilized and stained for GFP using A647-anti-GFP, counterstained with DAPI and imaged with 40X objective using confocal microscopy. Scale bars= 50µm.The mean GFP signal intensity was quantified from the images using ImageJ and normalized to the amount of DAPI^+^ nuclei (right panel). Anova fisher LSD (n=3).


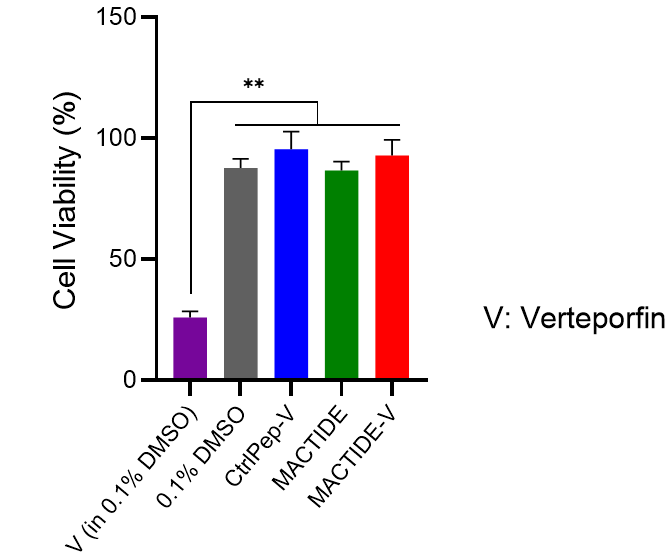


**Figure S5. In vitro cell viability in BMDM**. Day 5 BMDM in 96-well plate were treated with 10 μM MACTIDE, Verteporfin conjugates or Verteporfin alone (dissolved in 0.1% DMSO) during 4 h, followed by washing and addition of fresh media. 48h later, 10 µL of MTT (5 mg/mL) in PBS were added to the cells and incubated at 37°C for 2.5 h. Then, the medium was carefully removed, 100 µL of DMSO were added to each well and the plate was shaken until all crystals were dissolved. Absorbance was read at 570 nm using a plate reader. Cell viability is expressed as percentage of the untreated group. Anova fisher LSD (*n*=3).


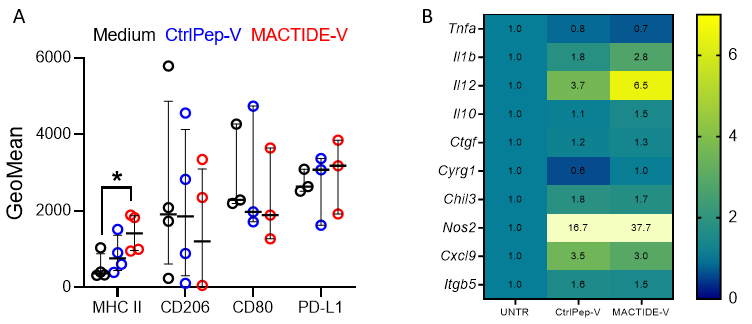


**Figure S6. In vitro effect of MACTIDE-V on B6 mice-derived BMDMs.** (A) Flow cytometry on BMDMs 48 h after treatment. GeoMean of MHC II in B6 BMDM, n=4 independent experiments. **p*≤0.05. **(B)** Heatmap of the mRNA expression of genes involved in the functional activation of BMDM, measured by real-time PCR 48 h after treatment in B6 BMDM, *n*=2 independent experiments.


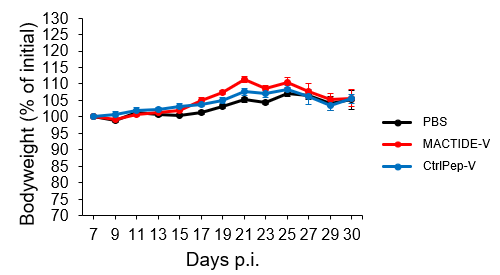


**Figure S7.** Bodyweights for treatment study of figure 7.


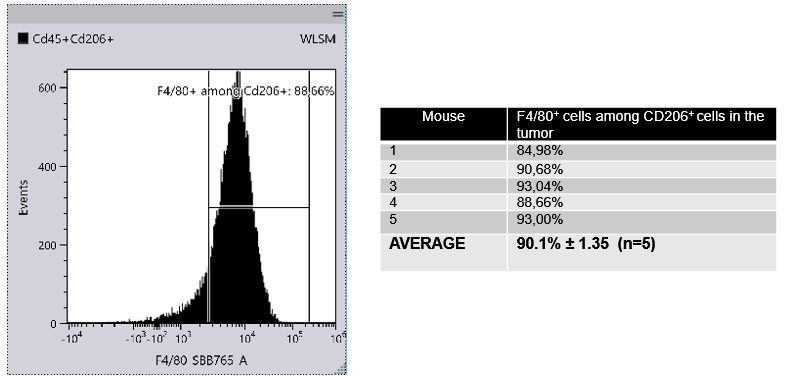


**Figure S8.** Representative flow cytometry plot of untreated 4T1.2 tumor showing F4/80+ cells among CD206+ cells in the tumor. Shown is a representative plot and on the left is the average from n=5 tumors.


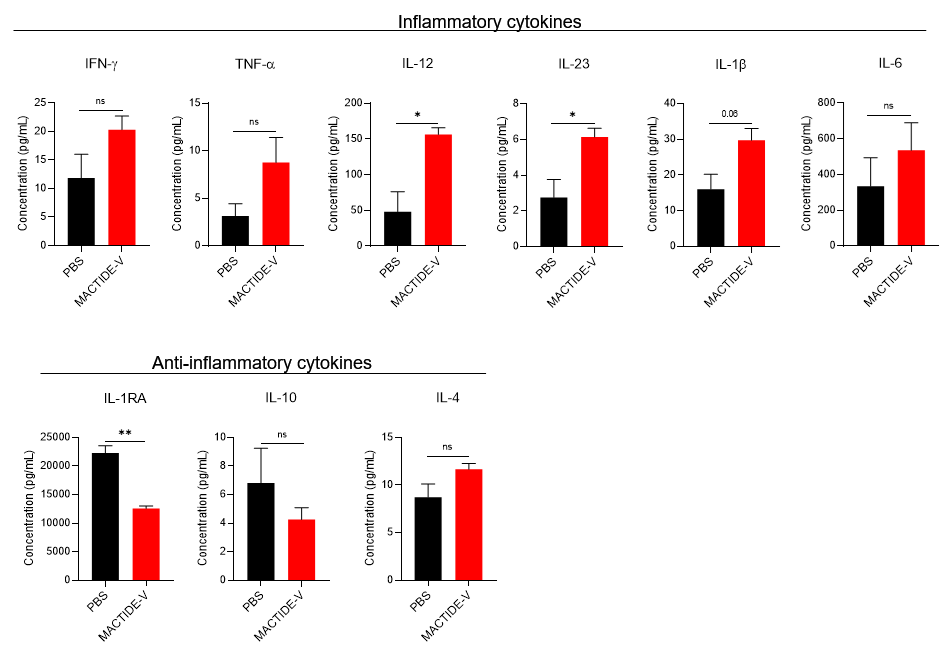


**Figure S9**. Quantification of cytokines from the supernatant of primary human macrophages treated with MACTIDE-V. Primary human macrophages (obtained from monocytes derived from human blood buffy coat) were incubated with 10µM MACTIDE-V for 4 h, followed by 2 washes with media, the cells were then placed in new media and cultured for an additional 48 h. Then, the supernatant was collected and analyzed for cytokine expression using a bead-based multiplexed cytokine panel assay (Biolegend, cat# 740502) following the manufacturer’s instructions. Anova Fisher LSD (n=3).

| **Analyte** | **MACTIDE-V**  **3 mg/Kg** | **Normal range** |
| --- | --- | --- |
| Alanine aminotransferase (U/I) | 52 ± 10.5 | 50-90 |
| Creatinine (μM) | 11.8 ± 1 | 10-36 |

**Table S1. Hepatic and renal toxicity analysed following one dose of MACTIDE-V.** Hepatic and renal toxicology serum levels of Crea and ALAT 48 h after i.p.-administration of MACTIDE-V (3 mg/kg) in healthy Balb/c mice (n=4). Normal ranges were obtained from Mouse Phenome Database by The Jackson Laboratory.


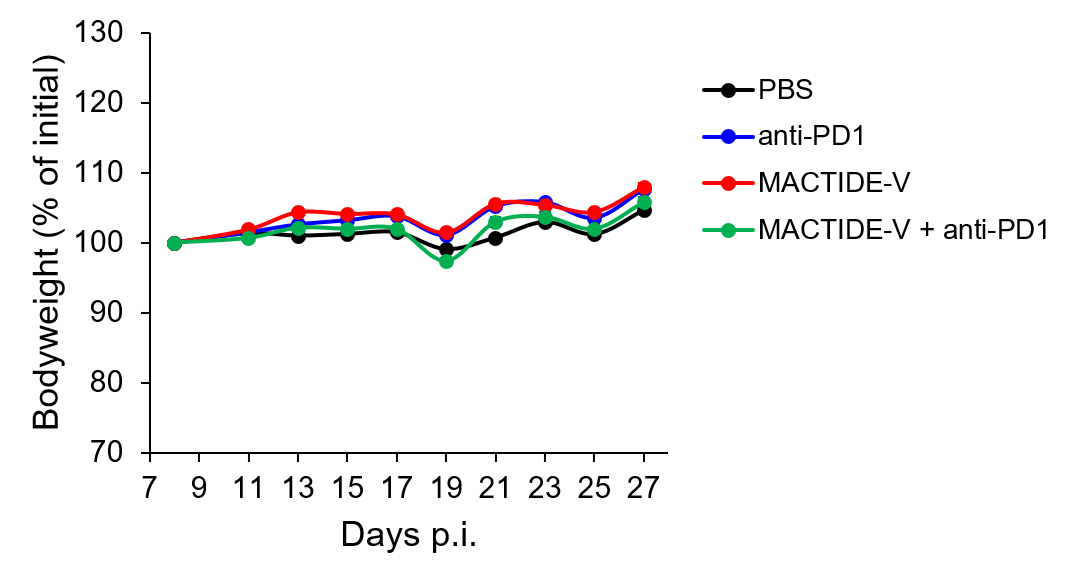


**Figure S10.** Bodyweights for treatment study of figure 8.


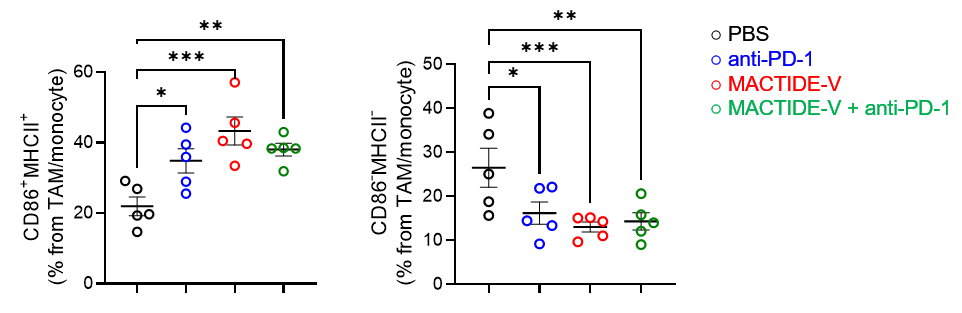


**Figure S11.** Flow cytometry of the orthotopic 4T1.2 tumors of Figure 8, *n*=5.
